# Mesenchymal stem cell-derived apoptotic vesicles ameliorate impaired ovarian folliculogenesis in polycystic ovary syndrome and ovarian aging by targeting WNT signaling

**DOI:** 10.7150/thno.94943

**Published:** 2024-05-27

**Authors:** Yu Fu, Manjin Zhang, Bingdong Sui, FeiFei Yuan, Wenbo Zhang, Yashuang Weng, Lei Xiang, Can Li, Longquan Shao, Yong You, Xueli Mao, Haitao Zeng, Di Chen, Meijia Zhang, Songtao Shi, Xuefeng Hu

**Affiliations:** 1 Fujian Key Laboratory of Developmental and Neural Biology & Southern Center for Biomedical Research, College of Life Sciences, Fujian Normal University, Fuzhou, Fujian 350117, China.; 2 Hospital of Stomatology, Guanghua School of Stomatology, Sun Yat-sen University, South China Center of Craniofacial Stem Cell Research, Guangdong Provincial Key Laboratory of Stomatology, Guangzhou, Guangdong 510055, China.; 3 Division of Cell, Developmental and Integrative Biology, School of Medicine, South China University of Technology, Guangzhou, Guangdong 510006, China.; 4 Stomatological Hospital, School of Stomatology, Southern Medical University, Guangzhou, Guangdong 510055, China.; 5 Research and Development Center for Tissue Engineering, The Fourth Military Medical University, Xi'an, Shaanxi 710032, China.; 6 Research Center for Computer-aided Drug Discovery, Shenzhen Institute of Advanced Technology, Chinese Academy of Sciences, Shenzhen 518055, China; 7 Reproductive Medicine Research Center, The Sixth Affiliated Hospital of Sun Yat-sen University, Guangzhou, Guangdong 510055, China.; 8 Department of Neurology, The Second Affiliated Hospital of Hainan Medical University, Haikou, Hainan 570013, China.; 9 International Center for Aging and Cancer (ICAC), Hainan Medical University. Haikou, Hainan 570013, China.; 10 Key Laboratory of Brain Science Research & Transformation in Tropical Environment of Hainan Province. Haikou, Hainan 570013, China.

**Keywords:** Apoptosis, Apoptotic vesicles, Ovarian homeostasis, Polycystic ovary syndrome (PCOS), Reproductive aging

## Abstract

**Rationale:** It has been emergingly recognized that apoptosis generates plenty of heterogeneous apoptotic vesicles (apoVs), which play a pivotal role in the maintenance of organ and tissue homeostasis. However, it is unknown whether apoVs influence postnatal ovarian folliculogenesis.

**Methods:** Apoptotic pathway deficient mice including Fas mutant (*Fas^mut^*) and Fas ligand mutant (*FasL^mut^*) mice were used with apoV replenishment to evaluate the biological function of apoVs during ovarian folliculogenesis. Ovarian function was characterized by morphological analysis, biochemical examination and cellular assays. Mechanistical studies were assessed by combinations of transcriptomic and proteomic analysis as well as molecular assays. *CYP17A1-Cre; Axin1^fl^*^/fl^ mice was established to verify the role of WNT signaling during ovarian folliculogenesis. Polycystic ovarian syndrome (PCOS) mice and 15-month-old mice were used with apoV replenishment to further validate the therapeutic effects of apoVs based on WNT signaling regulation.

**Results:** We show that systemic administration of mesenchymal stem cell (MSC)-derived apoptotic vesicles (MSC-apoVs) can ameliorate impaired ovarian folliculogenesis, PCOS phenotype, and reduced birth rate in *Fas^mut^* and *FasL^mut^* mice. Mechanistically, transcriptome analysis results revealed that MSC-apoVs downregulated a number of aberrant gene expression in *Fas^mut^* mice, which were enriched by kyoto encyclopedia of genes and genomes (KEGG) pathway analysis in WNT signaling and sex hormone biosynthesis. Furthermore, we found that apoptotic deficiency resulted in aberrant WNT/β-catenin activation in theca and mural granulosa cells, leading to responsive action of dickkopf1 (DKK1) in the cumulus cell and oocyte zone, which downregulated WNT/β-catenin expression in oocytes and, therefore, impaired ovarian folliculogenesis *via* NPPC/cGMP/PDE3A/cAMP cascade. When WNT/β-catenin was specially activated in theca cells of *CYP17A1-Cre; Axin1^fl^*^/fl^ mice, the same ovarian impairment phenotypes observed in apoptosis-deficient mice were established, confirming that aberrant activation of WNT/β-catenin in theca cells caused the impairment of ovarian folliculogenesis. We firstly revealed that apoVs delivered WNT membrane receptor inhibitor protein RNF43 to ovarian theca cells to balance follicle homeostasis through vesicle-cell membrane integration. Systemically infused RNF43-apoVs down-regulated aberrantly activated WNT/β-catenin signaling in theca cells, contributing to ovarian functional maintenance. Since aging mice have down-regulated expression of WNT/β-catenin in oocytes, we used MSC-apoVs to treat 15-month-old mice and found that MSC-apoVs effectively ameliorated the ovarian function and fertility capacity of these aging mice through rescuing WNT/β-catenin expression in oocytes.

**Conclusion:** Our studies reveal a previously unknown association between apoVs and ovarian folliculogenesis and suggest an apoV-based therapeutic approach to improve oocyte function and birth rates in PCOS and aging.

## Introduction

Apoptosis is a physiological and autonomous mode of programmed cell death (PCD) that contributes to routine cell turnover in organisms and plays a fundamental role in development, physio-pathogenesis and aging 1, 2. As apoptosis is executed, cells generate plentiful membrane-bound apoptotic compartments, previously known as apoptotic bodies (apoBDs); in addition, a great deal of evidence has recently revealed that smaller vesicular products called apoptotic vesicles (apoVs) are also produced during apoptotic cell disassembly 3, 4. Depending on their unique production mechanism, apoVs are capable of encapsulating various functional components of their parent cells, including nucleic acids, proteins and lipids, contributing to intercellular communication and maintenance of the whole body's homeostasis 5, 6. Notably, apoVs exhibit molecular properties and physiological characteristics quite distinct from exosomes and microvesicles, which lead to their distinctive targeting and functional modulation in specific physiological and pathological contexts 5. With growing acknowledgement of apoVs' uniquely beneficial properties, a series of pre-clinical studies have revealed that apoVs, particularly those derived from mesenchymal stem cells (MSCs), hold great therapeutic potential in wound repair, tissue regeneration and immunomodulation 7-11. However, it is unknown whether apoptosis and its metabolite apoVs regulate ovarian folliculogenesis.

The ovary is well recognized as a vital organ for female gametogenesis and sex hormone production. It has developed unique and tightly controlled cyclic structure and regulative mechanisms in response to follicle growth, steroidogenesis, oocyte maturation, ovulation, and corpus luteum formation 12. From fetal development to adulthood, apoptosis has long been recognized as a necessary part of female reproductive systems, playing an essential role in endogenous mechanisms for ovarian functional execution including germ cell depletion, follicular atresia and corpus luteum regression 13, 14. In pathological contexts, accumulating studies have documented that ovarian local apoptosis, particularly occurring in granulosa cells (GCs) and oocytes, is extensively involved in progression of ovarian diseases such as PCOS, premature ovarian failure (POF), ovarian aging, and ovarian cancer 15-17. However, whether systemic apoptosis and circulating apoptotic vesicles contribute to ovarian homeostasis and pathological development remains elusive.

In this study, we firstly show that ovarian polycystic-like changes and oocyte damage develop in apoptosis-deficient mice. MSC-apoV infusion effectively ameliorates these ovarian dysfunctions. To decipher the mechanism of how MSC-apoVs exert therapeutic effects, we performed transcriptome and bioinformatics analyses, which determined MSC-apoVs reduced activity in critical genes across several biological process in apoptosis-deficient mice, particularly in WNT signaling pathway. Further evidence reveals that WNT/β-catenin signaling in theca cells (TCs) is over-activated in apoptosis-deficient mice, which reactively activates DKK1 expression, leading to downregulation of WNT/β-catenin signaling in oocytes and thus impairment of ovarian folliculogenesis. Furthermore, we established WNT/β-catenin specially activated in theca cells of *CYP17A1-Cre; Axin1^fl^*^/fl^ mice to confirm the aberrant activation of WNT/β-catenin causing impaired ovarian phenotype. Intriguingly, we found apoVs inherit WNT membrane receptor inhibitor protein RNF43 from MSCs and transfer it to down-regulate aberrantly activated WNT/β-catenin signaling in recipient theca cells *via* unique vesicular-to-cell membrane communication mechanisms, further recovered whole follicle WNT signaling unbalance to ameliorate impaired ovarian folliculogenesis, PCOS phenotype, and reduced birth rate in apoptotic pathway deficient mice. Furthermore, we showed that MSC-apoV infusion ameliorated the PCOS phenotype in dehydroepiandrosterone (DHEA)-induced PCOS mice *via* rescuing impaired WNT/β-catenin signaling in the ovary. More importantly, apoV infusion significantly improves the ovarian function and fertility in aging mice through maintaining WNT/β-catenin homeostasis in oocytes.

## Results

### Apoptotic deficiency impairs ovarian folliculogenesis

**Figure 1:**
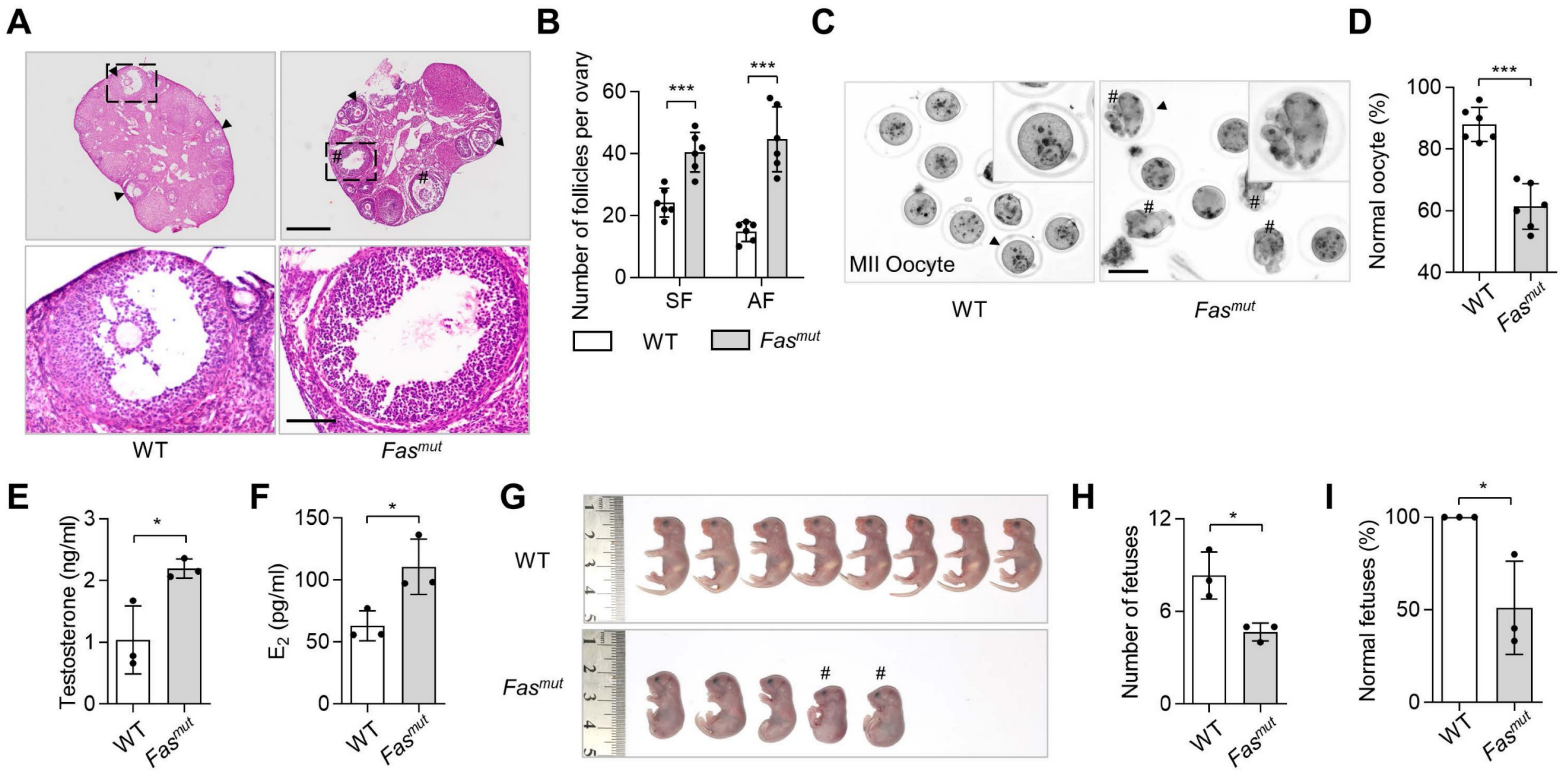
** Apoptosis-deficient *Fas^mut^* mice show ovarian dysfunction.** (**A**) Representative H&E staining of ovarian tissues. Lower panels are higher magnification views of the boxed regions in the above H&E staining images. Black arrowheads indicate antral follicles (AF). Pound signs indicate polycystic-like follicles. Scale bars, 500 μm for lower magnification, 100 μm for higher magnification. (**B**) The number of secondary follicles (SF) and AF in ovary. *N* = 6. (**C**) Gross appearance of meiosis II (MII) oocytes. Pound signs indicate abnormal oocytes. Black arrowheads indicate higher magnification views. Bar = 50 μm. (**D**) The ratio of normal MII oocytes/total oocytes. *N* = 6. (**E** and** F**) Enzyme linked immunosorbent assay (ELISA) examination for testosterone and estradiol (E_2_) level of serum. *N* = 3. (**G**) Gross appearance of fetuses from wildtype (WT) and *Fas^mut^* mice. Pound signs indicate deformed fetuses. (**H** and **I**) Number of fetuses and ratio of normal fetuses/total fetuses. *N* = 3. Error bars are means ± SD. Data were analyzed using two-tailed Student's unpaired *t* test for two-group comparison. NS, not significant. *P < 0.05, **P < 0.01, ***P < 0.001.

To investigate the role of apoptosis in ovarian homeostasis, we used two apoptotic pathway deficient mouse models, respectively the Fas mutant (*Fas^mut^*) and FasL mutant (*FasL^mut^*) mice 18, 19. FasL/Fas pathway have been widely studies as extrinsic apoptotic pathway triggering apoptosis execution, of which biomolecular mutant caused apoptosis deficiency 11. These two models both show excessive antral follicles (AF) and polycystic ovary syndrome (PCOS) phenotypes when compared to WT mice (Figure 1A-B and Figure S1A-B), along with significantly reduced levels of circulatory apoVs (Figure S2A). To explore whether fertility was impaired in apoptosis-deficient mice, we assessed oocyte quality and mating trials in *Fas^mut^* and *FasL^mut^* mice and found significant abnormalities of meiosis II (MII) oocytes after superovulation (Figure 1C-D and Figure S1E-F). Enzyme linked immunosorbent assay (ELISA) examination showed that the levels of testosterone and estradiol (E_2_) were elevated in *Fas^mut^* and *FasL^mut^* mice (Figure 1E-F and Figure S1C-D). These mice therefore met the Rotterdam criteria established in 2003 20, according to which PCOS can be diagnosed by the presence of polycystic-like histology together with elevated testosterone levels. Furthermore, the number of fetuses was obviously reduced in *Fas^mut^* mice (Figure 1G-H). Intriguingly, the gross appearance of fetuses of *Fas^mut^* mice showed extensive deformation, such as reduced body size, limb atrophy, and tail bending, as well as skeletal reduction and skull loss (Figure 1G-I and Figure S1G-I). Collectively, these findings suggest that apoptotic deficiency causes ovarian impairments.

### ApoV infusion ameliorates ovarian impairments in *Fas^mut^* and *FasL^mut^* mice

**Figure 2:**
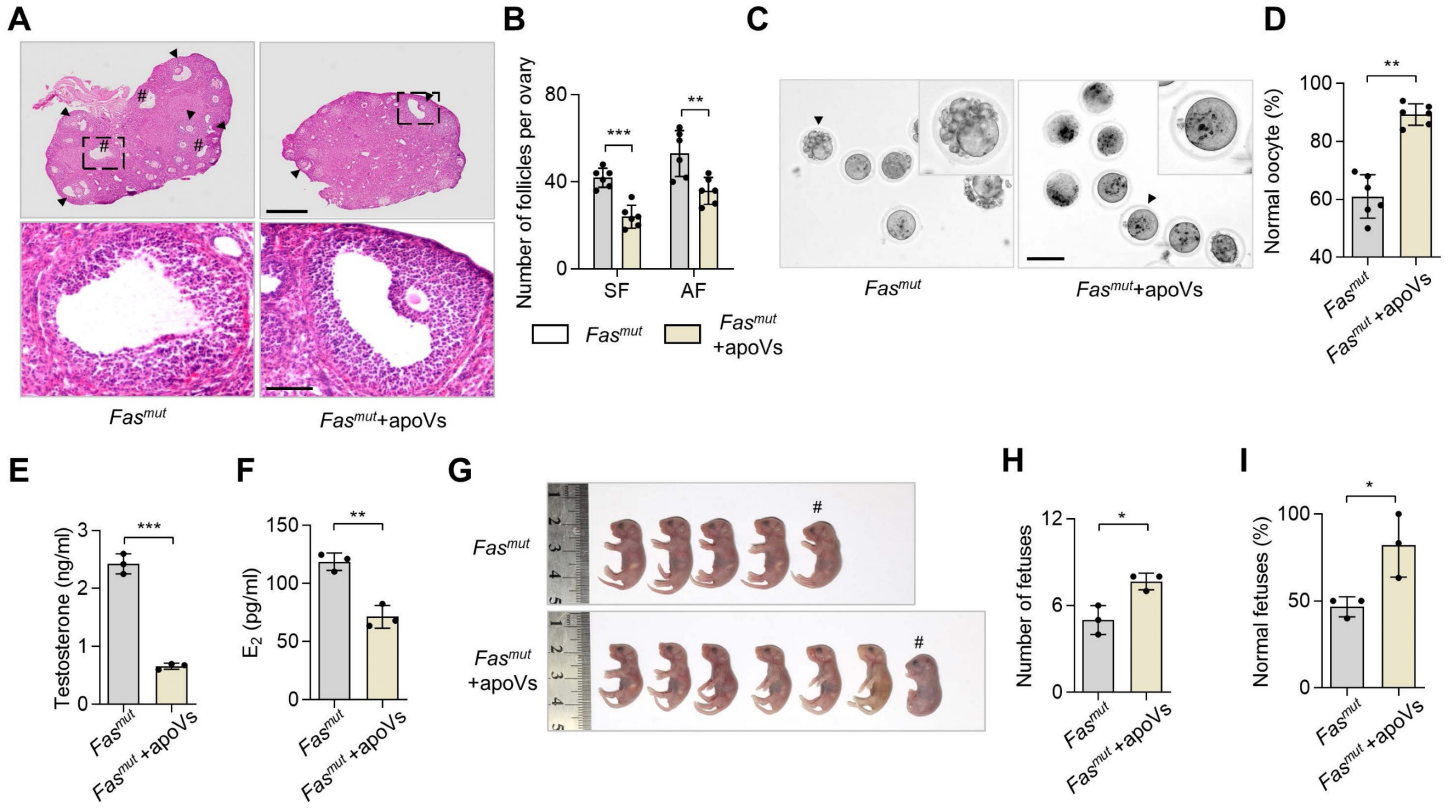
** MSC-apoV infusion ameliorates ovarian dysfunction in *Fas^mut^* mice.** (**A**) Representative H&E staining of ovarian tissues. Lower panels are higher magnification views of the boxed regions in the above H&E staining images. Black arrowheads indicate antral follicles (AF). Pound signs indicate polycystic-like follicles. Scale bars, 500 μm for lower magnification, 100 μm for higher magnification. (**B**) The number of SF and AF in ovary. *N* = 6. (**C**) Gross appearance of meiosis II (MII) oocytes. Pound signs indicate abnormal oocytes. Black arrowheads indicate higher magnification views. Scale bar, 50 μm. (**D**) The ratio of normal MII oocytes/total oocytes. *N* = 6. (**E** and **F**) ELISA for testosterone and estradiol (E_2_) level of serum. *N* = 3. (**G**) Gross appearance of fetuses from *Fas^mut^* mice with or without apoV injection. Pound signs indicate deformed fetuses. (**H** and **I**) Number of fetuses and ratio of normal fetuses/total fetuses. *N* = 3. Error bars are means ± SD. Data were analyzed using two-tailed Student's unpaired *t* test for two-group comparison. NS, not significant. *P < 0.05, **P < 0.01, ***P < 0.001.

Next, we investigated whether systemic infusion of MSC-derived apoVs (MSC-apoVs) could restore impaired ovarian function in *Fas^mut^* and *FasL^mut^* mice. We obtained apoVs from staurosporine (STS)-induced MSCs through sequential centrifuging (Figure S3A). The morphology of MSCs showed significant alteration after 8 h of STS induction (Figure S3B). TUNEL assay results indicated typical apoptotic responses (Figure S3C). Transmission electron microscopy (TEM), nanoparticle track analysis (NTA), and cytometric analysis were used to confirm the size, zeta potential and, surface marker profiles of the apoVs as previously described (Figure S3D-G) 5, 10. Then, we collected apoVs from 1 × 10^6^ MSCs and injected them into *Fas^mut^* and *FasL^mut^* mice *via* the tail vein. We found that MSC-apoV infusion effectively recovered impaired ovarian function in *Fas^mut^* and *FasL^mut^* mice, particularly with respect to the polycystic phenotype as well as the number of secondary follicles (SF) and AF (Figure 2A-B and Figure S1A-B). The gross appearance of MII oocytes indicated that the impairments of MII oocytes were ameliorated by MSC-apoV infusion in *Fas^mut^* and *FasL^mut^* mice (Figure 2C-D and Figure S1E-F). ELISA examination showed testosterone and E_2_ levels were rescued in *Fas^mut^* and *FasL^mut^* mice after MSC-apoV infusion (Figure 2E-F and Figure S1C-D). Furthermore, the number of fetuses and fetal abnormalities were obviously ameliorated in *Fas^mut^* mice after MSC-apoV infusion (Figure 2 G-I and Figure S1G-I). Taken together, these results indicate that MSC-apoV infusion offers effective therapeutic effects in apoptosis deficient mice to ameliorate impaired ovarian function.

### MSC-apoVs down-regulates aberrantly activated WNT/β-catenin signaling in theca cells

**Figure 3:**
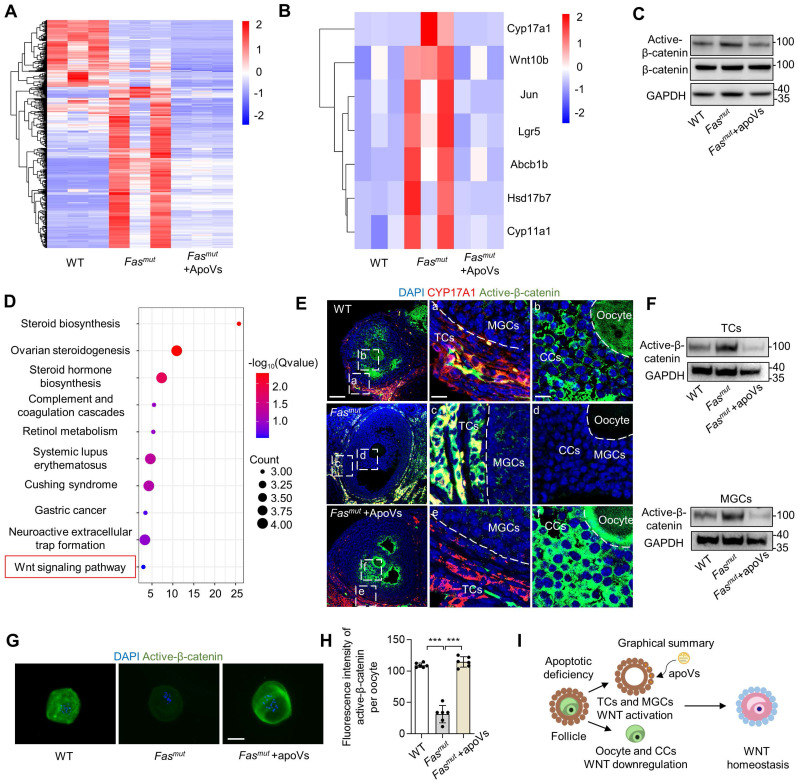
** Systemically infused MSC-apoVs rescue altered ovarian WNT/b-catenin signaling.** (**A**) Clustered heatmap detailing of DEGs in WT, *Fas^mut^* and *Fas^mut^* +apoVs mice. (Log_2_(fold change) > 1.0 and < -1.0, P value < 0.05) (**B**) DEG heatmap in in WT, *Fas^mut^* and *Fas^mut^* +apoVs mice. (Log_2_(fold change) > 1.0 and < -1.0, P value < 0.05) (**C**) Western blot analysis showing active β-catenin in the whole ovary. (**D**) KEGG pathway enrichment analysis of the intersection of up-regulated gene (WT vs *Fas^mut^*) and down regulated gene (*Fas^mut^* vs *Fas^mut^*+apoVs, Log_2_(fold change) > 1.8 and < -1.8). The Y-axis represents KEGG terms and the X-axis represents fold enrichment. (**E**) Immunofluorescent staining showing co-staining of TC-specific marker CYP17A1 and active β-catenin in the ovary. Right panels are higher magnification views of the left boxed regions, highlighting TC and mural granular cell (MGC) regions (a, c, e), as well as oocyte and cumulus cell (CC) regions (b, d, f). White dashed lines are used to divide cells in different regions. Scale bars, 50 μm for lower magnification, 10 μm for higher magnification. (**F**) Western blot analysis showing that active β-catenin levels in *in vitro* cultured TCs and MGCs. (**G**) Immunofluorescent staining of active β-catenin levels in germinal vesicle breakdown (GVBD) oocytes. Bar = 50 μm. (**H**) Quantification of fluorescence intensity of active β-catenin per oocyte showing significant differences. *N* = 6. (**I**) Graphical summary illustrates MSC-apoVs located in ovarian TCs and rescued WNT/β-catenin signaling dysfunction in follicles under apoptotic deficiency contexts. Error bars are means ± SD. Data were analyzed two-tailed Student's unpaired *t* test for two-group comparison or using one-way analysis of variance (ANOVA) with Tukey's test. NS, not significant. *P < 0.05, **P < 0.01, ***P < 0.001.

The above evidence encouraged us to investigate how apoVs regulate ovarian function. RNA-seq analysis was conducted to elucidate the mechanistic role of MSC-apoVs in the ovary of *Fas^mut^* mice. Through heatmaps (Figure 3A), we showed changes in 492 differentially expressed genes (DEGs). Notably, MSC-apoVs reduced a number of aberrantly upregulated gene expression in *Fas^mut^* mice, including WNT signaling targeting genes (Wnt10b, Jun, Lgr5 and Abcb1b) and sex hormone biosynthesis genes (Cyp17a1, Cyp11a1 and Hsd17b7, Figure 3B). Furthermore, we identified 162 DEGs (Log_2_(fold change) > 1.8 or < -1.8) upregulated in the WT vs *Fas^mut^* group while downregulated in the *Fas^mut^* vs *Fas^mut^*+apoVs group (Figure S4E). Based on these 162 DEGs, we performed kyoto encyclopedia of genes and genomes (KEGG) pathway analysis and gene ontology (GO) analysis, which revealed that MSC-apoVs altered expression of genes in *Fas^mut^* mice ovaries associated with signaling transduction and regulation of sex hormone biosynthesis, such as “Wnt signaling”, “Steroid biosynthesis”, “Ovarian steroidogenesis” and “Steroid hormone biosynthesis” (Figure 3D and Figure S4F). According to the RNA-seq results, we noticed that WNT signaling contribute to the regulative mechanisms of MSC-apoVs in *Fas^mut^* mice. As previously reported, WNT/β-catenin signaling was extensively changed in multiple organs in *Fas^mut^* mice, such as bone, skin, and kidney, known to be involved in organism functional regulation 10, 11, 21, 22. Therefore, western blot analysis was performed and showed that WNT/β-catenin signaling was abnormally overactivated in the ovaries of *Fas^mut^* mice, which could be rescued by MSC-apoV infusion (Figure 3C).

Furthermore, we sought to determine the biodistribution and physiological function of organismal apoVs. We found that systemically infused PKH26-labeled MSC-apoVs mainly concentrated in ovarian TCs, but not in granular cells (GCs) or oocytes (Figure S4A). To avoid membrane adhesion of PKH26 dye, we further used the luminogen DCPy with aggregation-induced emission characteristics (AIEgen) for mitochondria-targeted labeling of apoVs 23. Tracing of AIE-gen apoVs indeed showed that systemically infused MSC-apoVs particularly homed to ovarian TCs (Figure S4B). There was no significant difference in the cellular uptake rate (%) measured using these two different labeling methods (Figure S4C). Then, we isolated TCs from MSC-apoV-infused mice as previously described (Figure S5A) 24. TCs are the only cell population that produces androgens in the ovary, and they specifically express the steroidogenic marker CYP17A1 and mesenchymal cell marker Gli1 (Figure 3E and Figure S5B) 25, 26. As previously reported, apoVs tend to enrich in MSCs to regulate local tissue homeostasis 10, 11. With this in mind, we investigated the properties of TCs and discovered that they express MSC markers and possess both clonal proliferative ability and osteogenic capacity (Figure S5C-E). Notably, a kidney capsule implantation assay showed that TCs also possess powerful adipogenic capacity *in vivo* (Figure S5F-H). Next, we co-cultured PKH26-labeled apoVs with TCs and found that MSC-apoVs were mainly enriched in the perinuclear region within the TCs (Figure S4D). These data suggest that TCs may be a type of MSC-like cell that preferentially uptake apoVs.

Then, we assumed TCs may be the key targeting site of apoVs, which contribute to the ovary WNT signaling regulation. IF staining results showed that active β-catenin was over-activated in TCs and mural granulosa cells (MGCs) in *Fas^mut^* and *FasL^mut^* mice, alongside reduced expression of active β-catenin in cumulus cells (CCs) and oocytes. (Figure 3E, Figure S4G, Figure S6E). Importantly, MSC-apoV infusion reduced the levels of active β-catenin in TCs and MGCs while it increased active β-catenin expression in CCs and oocytes of *Fas^mut^* and *FasL^mut^* mice (Figure 3E, Figure S4G, Figure S6E). To further validate the effects of apoVs, we isolated TCs, MGCs and oocytes from *Fas^mut^* mice after MSC-apoV infusion. Western blot and IF staining showed that MSC-apoV infusion effectively rescued the altered active β-catenin expression in TCs, MGCs and oocytes (Figure 3F-H). These results collectively indicate that circulatory apoVs can home to ovarian TCs to maintain follicular WNT/β-catenin signaling homeostasis.

### WNT/β-catenin activation in theca cells causes reactively activated DKK1 expression in oocytes to down-regulate WNT/β-catenin

**Figure 4:**
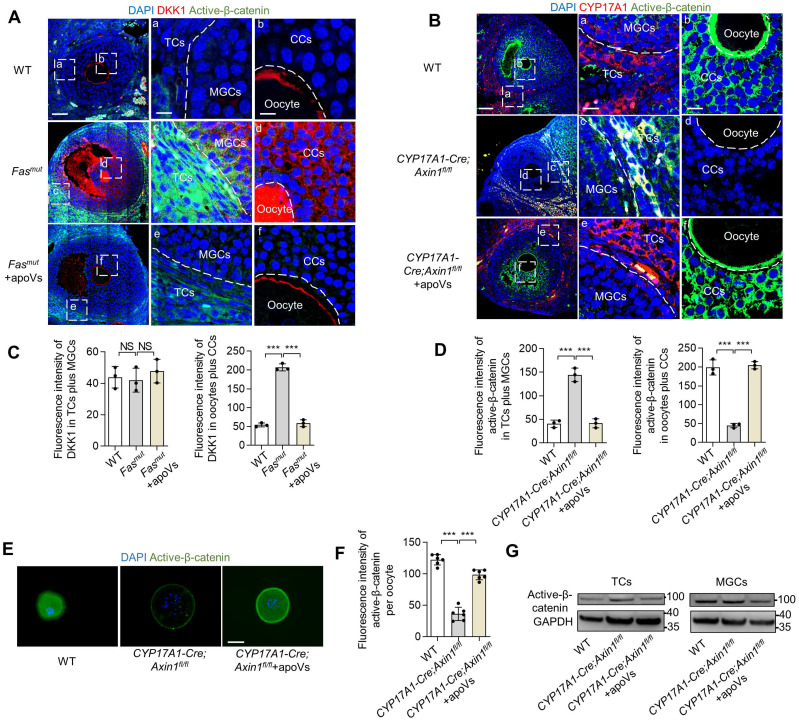
** WNT/β-catenin activation in theca cells causes reactively activated DKK1 expression in oocytes to down-regulate WNT/β-catenin.** (**A**) Immunofluorescent staining showing co-staining of WNT inhibitor protein DKK1 and active β-catenin in the ovaries of *Fas^mut^* and MSC-apoV-treated *Fas^mut^* mice. Right panels are higher magnification views of the left boxed regions, highlighting TC and MGC regions (a, c, e), as well as oocyte and CC regions (b, d, f). White dashed lines are used to divide cells in different regions. Scale bars, 50 μm for lower magnification, 10 μm for higher magnification. (**B**) Immunofluorescent staining showing co-staining of TC-specific marker CYP17A1 and active β-catenin in the ovary of *CYP17A1-Cre; Axin1^fl/fl^* mice. Right panels are higher magnification views of the left boxed regions, highlighting TC and MGC regions (a, c, e), as well as oocyte and CC regions (b, d, f). White dashed lines are used to divide cells in different regions. Scale bars, 50 μm for lower magnification, 10 μm for higher magnification. (**C**) Quantification of fluorescence intensity of DKK1 (red) in TCs plus MGCs and oocytes plus CCs.* N* = 3. (**D**) Quantification of fluorescence intensity of active β-catenin (green) in TCs plus MGCs and oocytes plus CCs.* N* = 3. (**E**) Immunofluorescent staining of active β-catenin level in GVBD oocytes. Scale bar, 50 μm. (**F**) Quantification of fluorescence intensity of active β-catenin per oocyte showing significant differences. *N* = 6. (**G**) Western blot analysis of active β-catenin levels in TCs and MGCs *in vitro* culture. Error bars are means ± SD. Data were analyzed using one-way analysis of variance (ANOVA) with Tukey's test. NS, not significant. *P < 0.05, **P < 0.01, ***P < 0.001.

We found systemically infused MSC-apoVs were enriched in TCs but nevertheless modulated WNT/β-catenin signaling throughout the whole follicles, a phenomenon which demonstrates the critical role of TCs in the regulation of ovarian WNT/β-catenin signaling homeostasis. We noticed with interest that WNT/β-catenin signaling was downregulated in oocytes of *Fas^mut^* mice. Since WNT/β-catenin signaling extensively contributes to the functional execution of oocytes 12, 27, 28, we deciphered how this down-regulation was achieved. We discovered that DKK1 was significantly elevated in CCs and oocytes in *Fas^mut^* mice compared to controls (Figure 4A). Importantly, MSC-apoV infusion restored normal DKK1 levels in the ovaries of *Fas^mut^* mice (Figure 4A). Accordingly, we inferred that reactively activated DKK1 in the CC and oocyte zone could contribute to the WNT/β-catenin signaling downregulation in oocytes. To validate that the altered WNT/β-catenin signaling in ovarian follicles was due to aberrant activation of active β-catenin in TCs, we established *CYP17A1-Cre; Axin1^fl^*^/fl^ mice, that specifically knocked out WNT signaling negative regulator Axin1 in theca cells to activate WNT/β-catenin (Figure S2B-C) and examined their active β-catenin levels. IF results showed that active β-catenin was elevated in ovarian TCs and MGCs of *CYP17A1-Cre; Axin1^fl^*^/fl^ mice (Figure 4B and D). Intriguingly, the active β-catenin level was reduced in the regions of cumulus cells (CCs) and oocytes, similar to what we observed in *Fas^mut^* mice (Figure 4B and D). IF staining further confirmed the reduction of active β-catenin expression in oocytes of *CYP17A1-Cre; Axin1^fl^*^/fl^ mice (Figure 4E-F). Importantly, MSC-apoV infusion restored normal WNT/β-catenin signaling in follicles of *CYP17A1-Cre; Axin1^fl^*^/fl^ mice (Figure 4B and D). Western blot and IF staining further confirmed that MSC-apoV infusion effectively rescued the WNT/β-catenin signaling in TCs, MGCs and oocytes of *CYP17A1-Cre; Axin1^f^*^l/fl^ mice (Figure 4E-G). Taken together, these findings indicate that aberrant WNT/β-catenin activation in theca cells causes responsive action of DKK1 in CCs and oocytes, leading to the WNT/β-catenin signaling downregulation in oocytes.

### Specific activation of WNT/β-catenin in TCs results in PCOS phenotype

**Figure 5:**
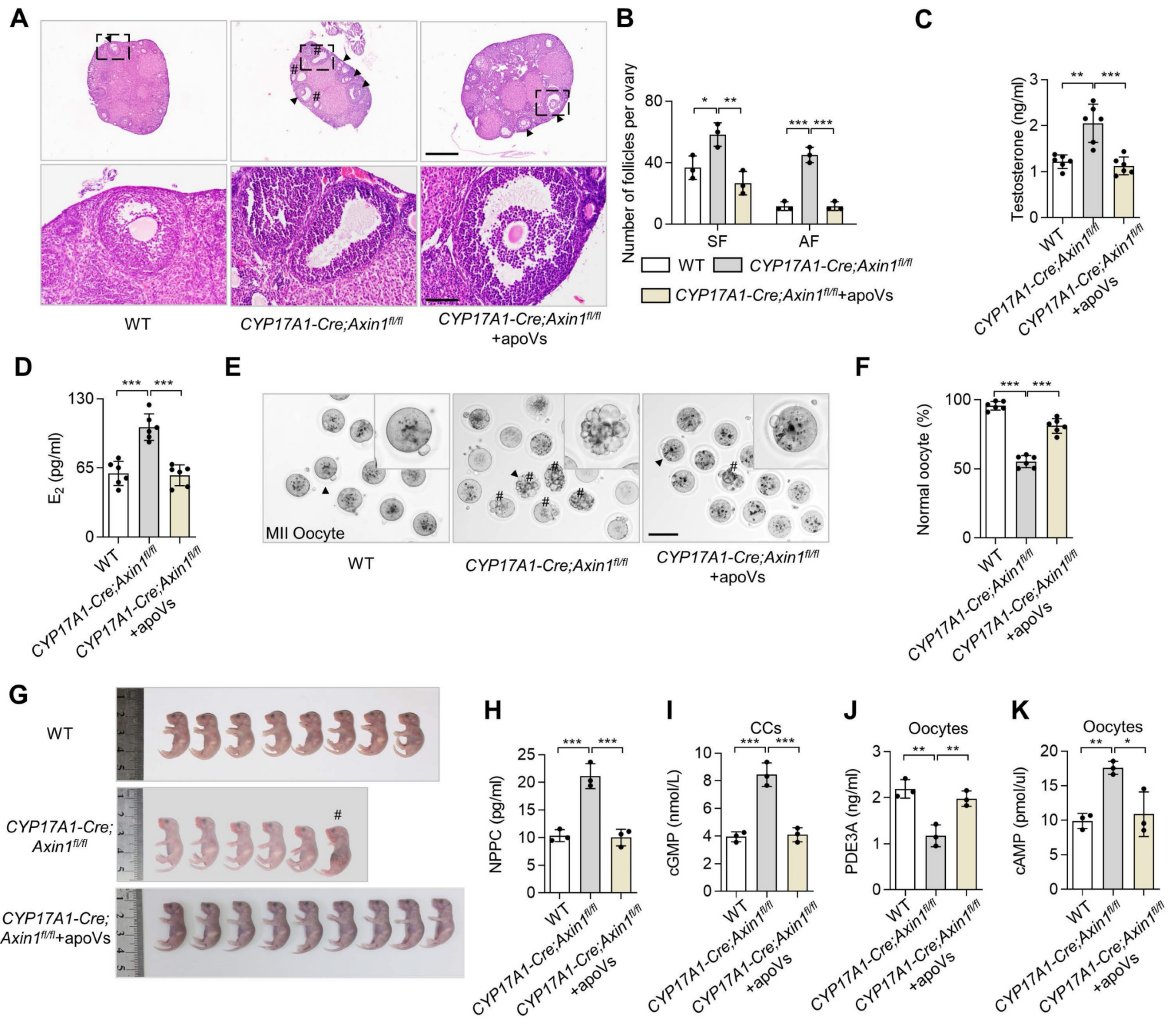
** Theca cell-specific WNT/β-catenin activation causes ovarian impairment.** (**A**) Representative H&E staining of ovarian tissues in *CYP17A1-Cre; Axin1^fl/fl^* mice, i.e. theca cell-specific WNT signaling overexpression mice. Lower panels are higher magnification views of the boxed regions in the above H&E staining images. Black arrowheads indicate AF. Pound signs indicate polycystic-like follicles. Scale bars, 500 μm for lower magnification, 100 μm for higher magnification. (**B**) The numbers of SF and AF in the ovaries. *N* = 3. (**C** and **D**) ELISA analysis showing the levels of testosterone and estradiol (E_2_) in serum. *N* = 6. (**E**) Gross appearance of MII oocytes. Pound signs indicate abnormal oocytes. Black arrowhead indicates higher magnification views. Scale bar, 50 μm. (**F**) The ratio of normal MII oocytes/total oocytes. *N* = 6. (**G**) Gross appearance of fetuses from *CYP17A1-Cre; Axin1^fl/fl^* mice with or without MSC-apoV injection. Pound signs indicate deformed fetuses. (**H**) ELISA analysis showing NPPC levels in MGCs. *N* = 3. (**I**) ELISA showing cGMP levels in CCs. *N* = 3. (**J** and **K**) ELISA showing PDE3A and cAMP levels in oocytes. *N* = 3. Error bars are means ± SD. Data were analyzed using one-way analysis of variance (ANOVA) with Tukey's test. NS, not significant. *P < 0.05, **P < 0.01, ***P < 0.001.

To further confirm that the aberrant activation of WNT/β-catenin in TCs resulting in downregulation of WNT/β-catenin in oocytes contribute to ovarian impairments, we examined the ovaries of *CYP17A1-Cre; Axin1^fl^*^/fl^ mice. Representative H&E staining of their ovarian tissues showed excessive SF and AF in the ovary (Figure 5A-B). Intriguingly, PCOS phenotypes were observed in *CYP17A1-Cre; Axin1^fl^*^/fl^ mice (Figure 5A-B). ELISA analysis showed that their testosterone and E_2_ levels were increased (Figure 5C-D). *CYP17A1-Cre; Axin1*^fl/fl^ mice also showed poor oocyte quality, as assessed by the gross appearance of MII oocytes and ratio of normal MII oocytes (Figure 5E-F). Furthermore, the number of fetuses was reduced in *CYP17A1-Cre; Axin1*^fl/fl^ mice (Figure 5G). Importantly, MSC-apoV infusion effectively ameliorated these ovarian impairments (Figure 5A-F). Taken together, these results indicate that altered follicular WNT/β-catenin signaling and impaired ovarian dysfunction are associated with activated WNT/β-catenin in ovarian TCs.

### MSC-apoV infusion ameliorates impaired oocytes *via* NPPC/cGMP/PDE3A/cAMP cascade

**Figure 6:**
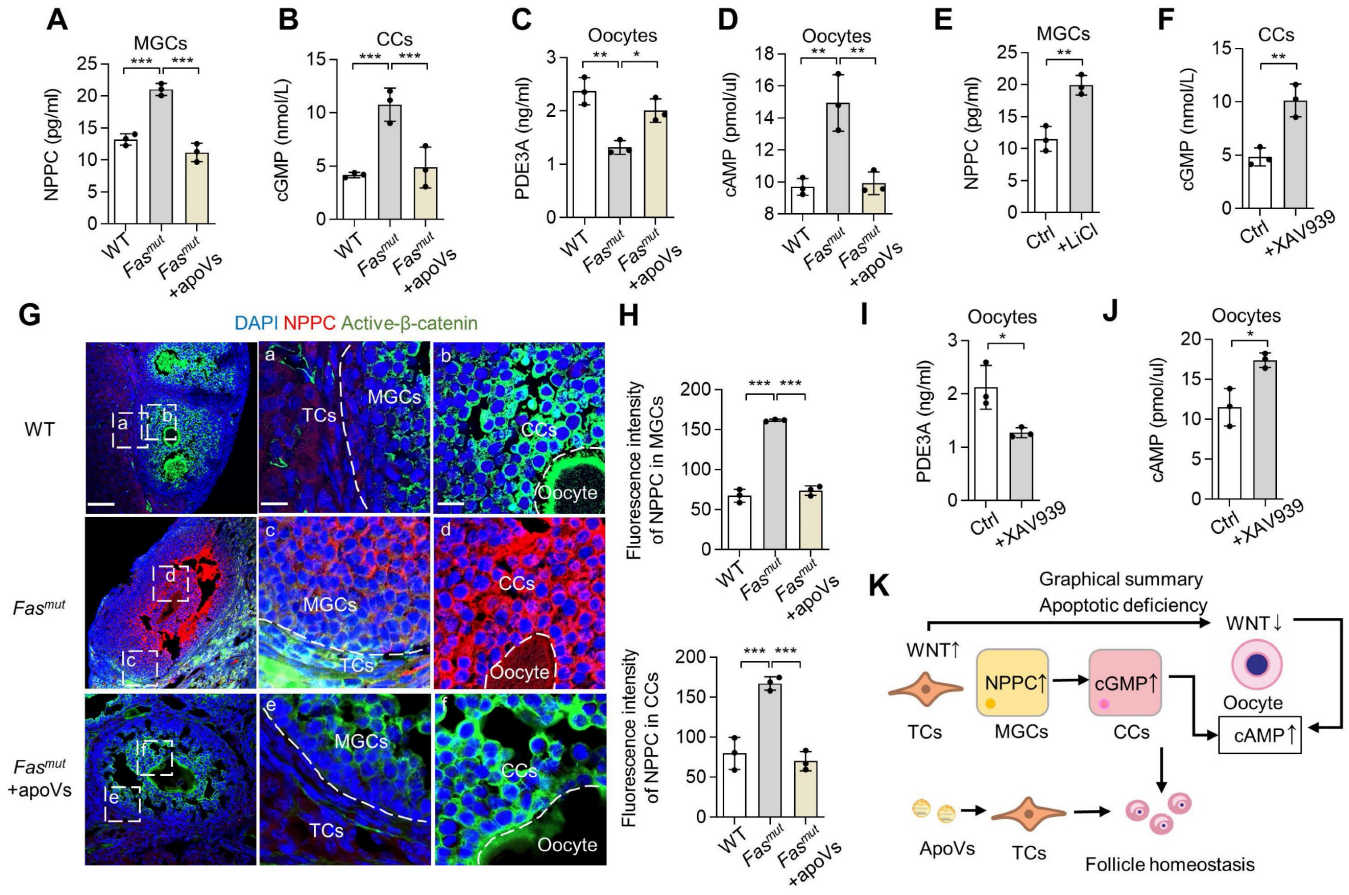
** NPPC/cGMP/PDE3A/cAMP cascade is recovered by MSC-apoV infusion.** (**A**) ELISA showing NPPC levels in MGCs. *N* = 3. (**B**) ELISA showing cGMP levels in CCs. *N* = 3. (**C** and **D**) ELISA showing PDE3A and cAMP levels in oocytes. *N* = 3. (**E**) ELISA showing NPPC levels in MGCs from WT mice after LiCl treatment. *N* = 3. (**F**) ELISA showing cGMP levels in CCs from WT mice after XAV939 treatment. *N* = 3. (**G and H**) Immunofluorescent co-staining of NPPC and active-β-catenin in the ovaries of *Fas^mut^* mice and MSC-apoV-treated *Fas^mut^* mice. Right panels are higher magnification views of the left boxed regions, highlighting TC and MGC regions (a, c, e), as well as oocyte and CC regions (b, d, f). White dashed lines are used to divide cells in different regions. Scale bars, 50 μm for lower magnification, 10 μm for higher magnification. (**I** and **J**) ELISA showing PDE3A and cAMP levels in oocytes of WT mice after XAV939 treatment. *N* = 3. (**K**) Graphical summary illustrating that WNT/β-catenin activation in MGCs triggers up-regulation of NPPC, leading to elevated levels of cGMP in CCs. Increased cGMP diffuses into oocytes to inhibit PDE3A activity and finally causes up-regulation of cAMP. On the other hand, down-regulation of WNT/β-catenin signaling in CCs and oocytes elevates the levels of cGMP and cAMP. Error bars are means ± SD. Data were analyzed two-tailed Student's unpaired *t* test for two-group comparison or using one-way analysis of variance (ANOVA) with Tukey's test. NS, not significant. *P < 0.05, **P < 0.01, ***P < 0.001.

We then evaluated how apoVs rescue WNT/β-catenin signaling homeostasis and ovarian function. According to the multicellular functional regulation mechanisms of apoVs, we naturally considered detecting functional pathways involved in the NPPC/cGMP/PDE3A/cAMP cascades in follicles 29. The NPPC/cGMP/PDE3A/cAMP cascade involves natriuretic peptide precursor C (NPPC) of MGCs interacting with receptor NPR2 on CCs to enhance cGMP levels, after which the cGMP diffuses into oocytes to inhibit phosphodiesterase 3A (PDE3A) activity and finally increase cAMP levels 29. cAMP is a key molecule in regulation of oocyte maturation, and high levels cause meiotic arrest 30. We found that NPPC levels of MGCs and cGMP levels of CCs were increased in *Fas^mut^* mice, both of which were rescued by MSC-apoV infusion (Figure 6A-B). We further isolated oocytes to examine their PDE3A and cAMP levels and found that PDEA3 was reduced while cAMP was elevated in *Fas^mut^* oocytes, both of which were recovered after MSC-apoV infusion (Figure 6C-D). To reveal whether the altered WNT/β-catenin signaling affects the NPPC/cGMP/PDE3A/cAMP cascades in ovaries, we treated MGCs with WNT activator lithium chloride (LiCl) and treated CCs and oocytes with WNT inhibitor XAV939 31, 32. ELISA results showed that NPPC levels in MGCs increased after LiCl treatment and cGMP levels in CCs increased after XAV939 treatment (Figure 6E-F). ELISA analysis further showed that PDE3A levels in oocytes decreased whereas cAMP levels in oocytes from WT mice increased after XAV939 treatment (Figure 6I-J). IF staining results showed that NPPC was over-activated in *Fas^mut^* MGCs along with increased expression of active β-catenin (Figure 6G-H). MSC-apoV infusion reduced the levels of NPPC in MGCs through modulating WNT/β-catenin of follicles (Figure 6G-H).

To further study whether this NPPC/cGMP/PDE3A/cAMP cascade disorder was caused by aberrant activation of WNT/β-catenin in TCs, we examined NPPC/cGMP/PDE3A/cAMP levels in the ovaries of *CYP17A1-Cre; Axin1^fl^*^/fl^ mice and found that the levels of NPPC and cGMP were increased in MGCs and CCs, and the PDE3A level was decreased while the cAMP level was increased in oocytes of *CYP17A1-Cre; Axin1^fl^*^/fl^ mice (Figure 5H-K). Notably, MSC-apoV infusion significantly improved the impaired NPPC/cGMP/PDE3A/cAMP cascade in *CYP17A1-Cre; Axin1^fl^*^/fl^ mice (Figure 5H-K). Collectively, these results revealed that MSC-apoV infusion rescued the NPPC/cGMP/PDE3A/cAMP cascade and ovarian function.

### MSC-apoV infusion ameliorates ovarian impairment *via* transfer of RNF43 in *Fas^mut^* mice

**Figure 7:**
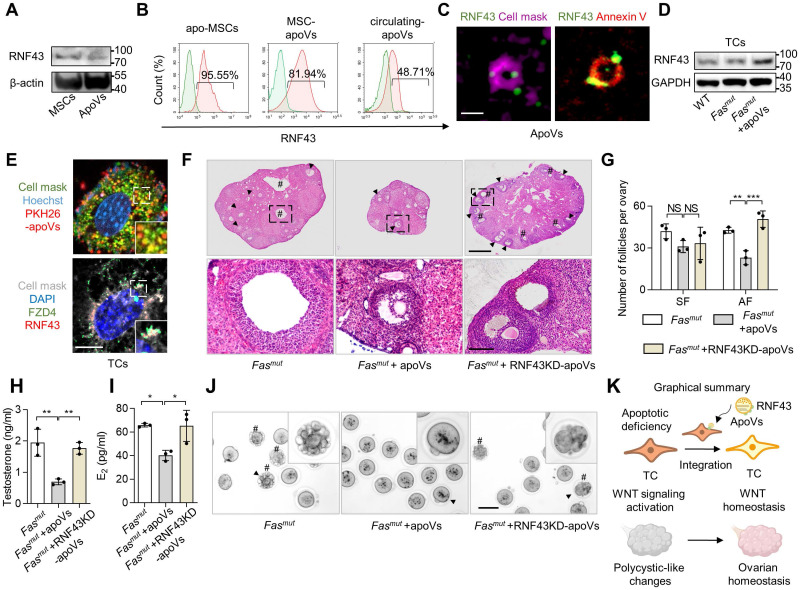
** RNF43 contributes to MSC-apoV-mediated ameliorate of ovarian dysfunction.** (**A**) Western blot analysis showing RNF43 expression in MSCs and MSC-apoVs. (**B**) Immunofluorescent staining showing MSC-apoV surface expression of RNF43. Bar = 500 nm. (**C**) Flow cytometry analysis showing RNF43 expression in MSCs and MSC-apoVs as well as circulating apoVs. (**D**) Western blot analysis showing RNF43 expression level in ovarian TCs when co-cultured with MSC-apoVs* in vitro*. (**E**) Immunofluorescent staining of exogenous MSC-apoVs (red) co-cultured with TCs. Higher magnification views are boxed regions, showing apoV integration with recipient TC membrane and interaction with WNT receptor FZD4 in TCs. Scale bar, 10 μm. (**F**) Representative H&E staining of ovarian tissues showing that apoVs derived from RNF43 knockdown (RNF43KD)-MSCs failed to ameliorate polycystic-like disorders in *Fas^mut^* mice. Black arrowheads indicate AF. Pound signs indicate polycystic-like follicles. Scale bar, 500 μm for lower magnification, 100 μm for higher magnification. (**G**) The numbers of SF and AF in ovaries. *N* = 3. (**H** and **I**) ELISA showing apoVs derived from RNF43KD-MSCs failed to rescue testosterone and E_2_ levels in *Fas^mut^* mice. *N* = 3. (**J**) Gross appearance of MII oocytes showing apoVs derived from RNF43KD-MSCs failed to ameliorate abnormal oocytes in *Fas^mut^* mice. Pound signs indicate abnormal oocytes. Black arrowheads indicate higher magnification views. Bar = 50 μm. (**K**) Graphical summary of RNF43 derived from MSC-apoVs rescuing aberrantly activated WNT/β-catenin signaling in TCs in the apoptotic deficiency context, while functionally rescuing polycystic-like disorders in *Fas^mut^* mice. Error bars are means ± SD. Data were analyzed using one-way analysis of variance (ANOVA) with Tukey's test. NS, not significant. *P < 0.05, **P < 0.01, ***P < 0.001.

Next, we aimed to decipher how apoVs safeguard against activated WNT/β-catenin signaling in TCs. A recent study showed the proteomic analysis of MSC-apoVs 5. In our re-analysis of this proteome, we found that enriched protein in apoVs were associated with “Endocrine system” within “Organismal systems” (Figure S3H). We further analyzed the proteins from “Endocrine system” using KEGG pathway enriched analysis, which indicated the involvement of apoVs in “Wnt signaling” (Figure S3I). Previous studies revealed that apoVs can activate WNT/β-catenin signaling in bone marrow MSCs and in the integumentary system *via* transferring mRNA and β-catenin 10, 11. Therefore, we began with the operating premise that MSC-apoVs rescued WNT/β-catenin signaling throughout the whole follicle in *Fas^mut^* mice *via* specific modulation of TCs. We discovered that MSC-apoVs not only packaged β-catenin, but also contained ring finger protein 43 (RNF43) (Fig 7A). RNF43 is a single-pass transmembrane E3 ligases, which specifically targets WNT receptor Frizzled 4 (FZD4) for ubiquitination and turnover, constituting a negative WNT feedback loop 33, 34. Flow cytometry analysis confirmed the expression of RNF43 in MSCs and MSC-apoVs as well as circulating apoVs (Figure 7B). The results of lattice structured illumination microscopy (SIM) further revealed that MSC-apoVs exhibited surface expression of RNF43 (Figure 7C). RNF43 expression levels in *Fas^mut^* ovarian TCs increased after co-cultured with MSC-apoVs (Figure 7D). In dissecting whether and how exogenous apoVs communicate with recipient TCs, we co-cultured PKH26-labeled MSC-apoVs with TCs and found that MSC-apoVs integrated with the TC membrane (Figure 7E). Then, we co-cultured RNF43 fluorescent-labeled MSC-apoVs with TCs, discovering the RNF43 of apoVs interacted with WNT receptor FZD4 of TCs (Figure 7E).

We further investigated whether RNF43 was indeed pivotal for mediating the therapeutic effects of MSC-apoVs in *Fas^mut^* mice by using RNF43- deficient apoVs derived from RNF43 siRNA knocking down (RNF43KD) MSCs (Figure S2D-E). We revealed that RNF43KD-apoVs failed to rescue excessive AF as well as PCOS phenotype in *Fas^mut^* mice (Figure 7F-G). ELISA analysis showed that RNF43KD-apoVs failed to rescue testosterone and E_2_ levels in *Fas^mut^* mice (Figure 7H-I). RNF43KD-apoVs also failed to ameliorate abnormal oocytes in *Fas^mut^* mice as assessed by the gross appearance of MII oocytes (Figure 7J). Briefly, these findings indicate that RNF43 is required for apoV-mediated ameliorate of impaired ovarian function in *Fas^mut^* mice.

### MSC-apoV infusion ameliorates DHEA-induced PCOS and ameliorates aging-associated ovarian impairment

**Figure 8:**
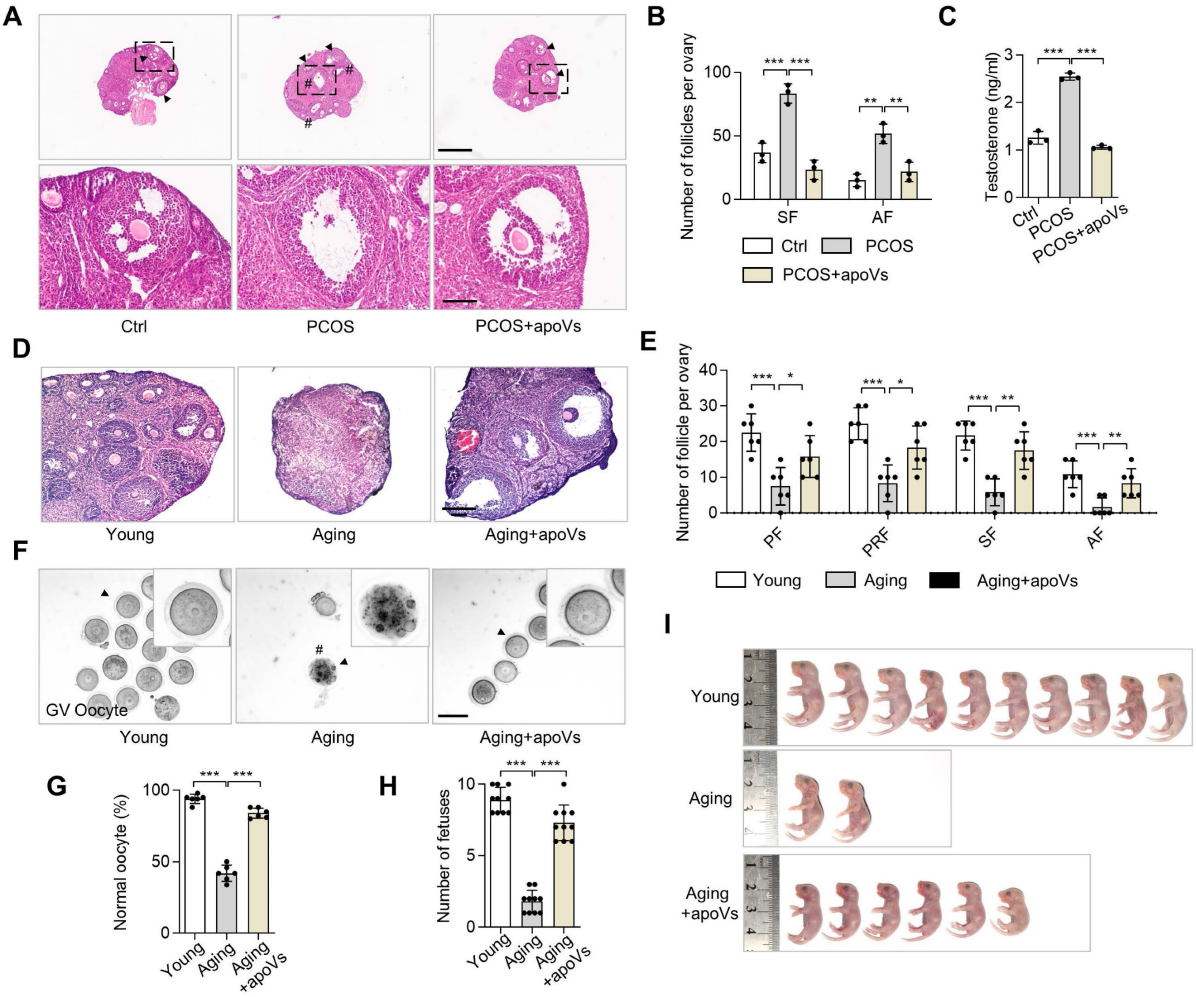
** MSC-apoV infusion rescues DHEA-induced PCOS and impairment of aging ovaries.** (**A**) Representative H&E staining of ovarian tissues in control group (Ctrl) mice as well as PCOS mice with or without apoV injection. Black arrowheads indicate AF. Pound signs indicate polycystic follicles. Scale bars, 500 μm for lower magnification, 100 μm for higher magnification. (**B**) The numbers of SF and AF in the ovaries. *N* = 3. (**C**) ELISA analysis showing the level of testosterone in serum. *N* = 3. (**D**) Representative H&E staining of ovarian tissues in young mice as well as aging mice with or without apoV injection. Scale bar, 100 μm. (**E**) The numbers of primordial follicles (PF), primary follicles (PRF), SF and AF in the ovaries. *N* = 6. (**F**) Gross appearance of germinal vesicle (GV) oocytes. Pound signs indicate abnormal oocytes. Black arrowheads indicate higher magnification views. Bar = 50 μm. (**G**) The ratio of normal GV oocytes/total oocytes. *N* = 6. (**H**) Number of fetuses. *N* = 10. (**I**) Gross appearance of fetuses from young and aging mice. Pound signs indicate deformed fetuses. Error bars are means ± SD. Data were analyzed using one-way analysis of variance (ANOVA) with Tukey's test. NS, not significant. *P < 0.05, **P < 0.01, ***P < 0.001.

Based on our findings that MSC-apoVs can ameliorate PCOS phenotypes, we considered whether apoV infusion could ameliorate dehydroepiandrosterone (DHEA)-induced PCOS mice. We administered DHEA *via* subcutaneous injections to induce PCOS phenotypes as previously described 35 and subsequently infused MSC-apoVs *via* the tail vein. Representative H&E staining of ovarian tissues in DHEA-induced PCOS mice showed MSC-apoV infusion effectively ameliorated the PCOS phenotypes, including polycystic changes, excessive SF and AF, and high testosterone (Figure 8A-C). Intriguingly, the active β-catenin level of PCOS mice was increased in the regions of TCs and was reduced in the CCs and oocytes, similar to what we observed in *Fas^mut^* mice (Figure S6D).

Given that our studies here focused largely on abnormalities of oocytes in multiple mouse models in pathological contexts accompanied by the down-regulation of WNT/β-catenin signaling in oocytes, we naturally hypothesized that decreased WNT signaling under physiological conditions could cause a reduction in oocyte quality and thus a reduction in fertility, as is associated with aging. Accumulating studies have demonstrated that WNT/β-catenin signaling deficiency is associated with pathological progression of the ovary 36-39. However, whether apoVs could improve the function of aging ovarian *via* regulating WNT/β-catenin signaling has not been explored. Accordingly, we investigated the ovarian status and fertility of 15-month-old mice with or without MSC-apoV infusion. IF staining and western blot analysis confirmed that WNT/β-catenin signaling was reduced in oocytes of aging ovaries (Figure S6A -C). MSC-apoV infusion elevated WNT/β-catenin expression in oocytes (Figure S6A-C). Representative H&E staining showed a decreased in the number of follicles in the ovaries of untreated aging mice, while MSC-apoV infusion effectively increased the number of follicles in aging mice (Figure 8D-E). Gross inspection of GV oocytes' appearance showed the reduced number and low quality of GV oocytes in aging mice, which were rescued after MSC-apoV infusion (Figure 8F-G). Importantly, we evaluated the fetal quantity of aging mice after MSC-apoV infusion and examined the progeny for any gross abnormalities. We found that MSC-apoV treatment significantly improved aging mouse fertility (Figure 8H-I). Taken together, our findings suggest a unique role of apoVs in ovarian folliculogenesis and therapeutic potential for MSC-apoVs in ovarian disorders.

## Discussion

Apoptosis is a prominent mode of programmed cell death and extensively contributes to maintenance of tissue homeostasis in multicellular organisms. As is traditionally known, apoptosis is closely correlated with immune regulation, because cells that undergo apoptosis are cleared by phagocytosis without inducing an immune reaction triggered by the escape of otherwise typically immunogenic cellular contents 40. Therefore, organisms like *Fas^mut^* mice with apoptotic deficiency develop spontaneous destructive immune disorders, such as lupus and rheumatoid arthritis 41. Although *Fas^mut^* mice have been reported to have abnormalities in ovarian function, this has been attributed to influences of autoimmune disease 42. *FasL^mut^* mice have also been documented to present impair oocyte competence, but this has been related to ovarian local apoptosis 43. According to the above recognition, whether systemic apoptosis involve in ovarian homeostasis and pathological development is unknown. With growing understanding of apoptosis during the last decade, it is now recognized that, far from being simply a “passive” form of cellular waste clearance in organisms, apoptosis involves more “active” release of intracellular materials, membrane contents and vesicles 5, 44. In this regard, accumulating studies have recently shown that apoptosis-derived vesicles (apoVs) possess extensive regulatory capacities in various organ systems and can exert therapeutic effects to ameliorate diverse impairments and diseases 7. However, whether apoptosis and apoVs contribute to ovarian homeostasis and pathological progression is unknown.

In this study, we discovered that apoptosis-deficient mice developed ovarian dysfunction and impaired fertility. Then, we infused exogenous MSC-apoVs into apoptosis-deficient mice and found the ovarian dysfunction was ameliorated. Mechanistically, the heatmap result indicated that the ovarian transcriptome level of apoptosis-deficient mice was increased on the most genes, demonstrating the activated transcriptome status under the disruption of the FasL/Fas signal in ovary. It is known that the reduction of FasL/Fas signal trigger the tumor development in mammary glands and gonads 45, 46, suggesting the consistency of FasL/Fas axis in homeostasis maintenance. We revealed MSC-apoVs extensively downregulate the aberrant transcriptome level of *Fas^mut^* mice, which contribute to the local modulation of WNT signaling (*Wnt10b*, *Jun*, *Lgr5* and *Abcb1b*) and sex hormone biosynthesis (*Cyp17a1*, *Cyp11a1* and *Hsd17b7*). As documented, *Cyp17a1* was involved in the synthesis of DHEA and androstenedione and *Hsd17b7* contributed to estradiol synthesis 47, of which the transcriptome changes in apoptosis-deficient mice with MSC-apoV replenishment was consistent with our Elisa results. Notably, we unexpectedly found the hypothalamic-pituitary originated hormone follicular stimulating hormone (FSH) and luteinizing hormone (LH) had no significant difference under the apoptosis-deficient settings (Data not shown). According to these results, the underlying cues between systematic apoptotic deficiency and ovariogenic hormone synthesis abnormity were established, which probably represented the new hormone regulatory mechanisms that being independent of hypothalamic-pituitary-ovarian axis. Furthermore, β-catenin has been proved as an essential transcriptional activator of aromatase gene expression 48. In view of the consistent tendency of transcriptome level in WNT signaling and sex hormone biosynthesis under apoptotic deficiency, the circulating apoV may be the indispensable mediator of the crosstalk between the two biological process.

Then, we dissected that ovarian TCs in an apoptosis-deficient setting present with aberrant WNT/β-catenin activation, which causes WNT/β-catenin signaling alteration throughout the whole follicle, particularly triggering down-regulation of WNT/β-catenin in oocytes. Based on transcriptome results and protein molecular experiments, apoV replenishment was proved to ameliorate the abnormity of WNT/β-catenin. To decipher the content of apoVs, we re-analyzed the proteomic results from previous study 5, and found the enriched proteins in apoVs were associated with “Endocrine system” within “Organismal systems. Through the KEGG pathway enriched analysis, the results indicated the involvement of apoVs in “Wnt signaling”. These data suggest the characterized biodistribution during apoptosis execution, the cell contents are enriched in apoVs contributing to specific signal pathway regulatory network. A recent RNA-seq results of MSC-apoVs also demonstrated the inheritance of apoVs from parental cells, reporting the important role of apoVs in regulating stem cell properties 49. Therefore, future studies will need to elaborate on the detailed RNA content of endogenous apoVs in modulating ovarian homeostasis and pathologies. In our researches, we found that systemically infused MSC-apoVs inherit the WNT membrane receptor inhibitor protein RNF43 from MSCs and transfer it to apoptosis-deficient ovarian TCs, rescuing WNT/β-catenin signaling *via* characteristic vesicular-to-cell membrane integration mechanisms. In previous study, we have reported the specific target of circulatory apoVs in liver homeostasis and regeneration, which mediated by the sugar recognition system 50. In this regard, how the endogenous apoVs target ovarian TCs, as our findings of apoV enrichment in the TCs of androgen synthesis, is interesting but not elucidated questions. The WNT/β-catenin signaling disorders of follicles in apoptosis-deficient mice lead to ovarian functional impairments by affecting the NPPC/cGMP/PDE3A/cAMP cascade. As the result of MSC-apoVs targeting TCs but regulating oocytes, we proposed the novel concept that TCs serve as “soil,” receiving apoVs that contribute to the functional maintenance of oocytes (“seeds”). To confirm our hypothesis, we established mice with ovarian impairments due to overexpression of WNT/β-catenin signaling specifically in the theca cells (*CYP17A1-Cre; Axin1^fl/fl^* mice), which revealed that WNT/β-catenin activation in TCs indeed caused a reduction in WNT/β-catenin expression in oocytes and ovarian dysfunction. MSC-apoV infusion was confirmed to recover the WNT/β-catenin signaling in oocytes in *CYP17A1-Cre; Axin1^fl/fl^* mice. In brief, we revealed a previously unrecognized regulatory link between circulatory apoVs and ovarian homeostasis.

The WNT signaling pathway controls innumerable physiological and pathological process throughout developmental and postnatal life of all animals 51. In the early gonadal primordium development, accumulating studies have documented that WNT signaling plays indispensable role in gonadogenesis, which determined two divergent fates contributing to formation of either testis or ovary 52. Furthermore, the WNT signaling molecules have emergingly been reported that extensively regulated reproductive function including folliculogenesis and steroidogenesis in postnatal ovarian 53. However, the mechanisms of how organisms modulated WNT signaling to orchestrate follicle function in postnatal ovary is still unknown. Here, we firstly revealed a unique mechanism of circulating apoVs inherit the WNT membrane receptor inhibitor protein RNF43 from MSCs and transfer it to integrate with ovarian TC membrane for WNT signaling down-regulation, presenting a novel regulative mode of vesicular-to-cell membrane communication that particularly distinguished with cell-vesicular internalization 11. According to these findings, RNF43 enriched apoVs may achieve new engineering approaches of vesicles for future targeting therapeutics of female reproductive diseases.

PCOS is the most common reproductive and endocrine disorder, it has become immensely prevalent, affecting 6-20% of women of reproductive age worldwide 54, 55. According to Rotterdam criteria established in 2003 20, PCOS is defined by the presence of two of the three following criteria: hyperandrogenism, polycystic ovaries and anovulation. In PCOS patients, ovarian anovulation is associated with a decreased number of resting follicles and an increased fraction of AF, so that TCs of PCOS patients convert androgens with great efficacy 56-58. Traditionally, intraovarian abnormalities in PCOS caused by hormone level alterations have been considered a major concern for pathological progression, mainly attributed to ovarian hormone secretory cell dysfunction, particularly in TCs 59, 60. However, the mechanisms by which TCs are regulated in PCOS pathological progression remain unclear. Here, we revealed previously unknown mechanisms involving systemic apoptosis in the regulation of TCs associated with pathological progression of PCOS. We unexpectedly discovered that the ovaries of apoptosis-deficient mice developed excessive AF and polycystic-like phenotypes as well as excessive androgen. According to the Rotterdam criteria, these mice exhibited PCOS. Notably, MSC-apoV infusion effectively ameliorated the PCOS phenotype. Furthermore, we found that MSC-apoVs can effectively treat DHEA-induced PCOS mice. These findings suggest a novel avenue of research into how systemic apoptosis is involved in the pathological progression of PCOS, and provide a possible apoV-associated clinical therapeutic for PCOS.

Ovarian aging is a worldwide issue, affecting millions of women 61. Clinically, fertility in women begins to decline after age of 37 years and it is difficult to sustain pregnancy after 45 years, marking the early stages of an inevitable phenomenon that culminates in menopause 61-63. How to overcome age-related infertility in women is a vital but unsolved clinical issue. To date, cryopreservation of eggs retrieved from the ovaries at a younger age for later use is a common way for aging women to proactively preserve their fertility, but it cannot reverse ovarian aging or general health issues that can be caused by aging-related ovarian dysfunction 64. The drug metformin has shown promise in preventing age-associated ovarian dysfunctions, but the mechanisms of its action are still unknown 65. MSC transplantation has also been considered as a promising therapeutic strategy to improve ovarian senescence, but diverse problems limit its application, such as immunogenicity and difficulties in storage and delivery 66. Recent research revealed that MSC-apoVs assemble multiple nuclear DNA repair enzymes and rescued the DNA damage of aging 67. However, how the apoVs being the effective and safe therapeutics to stem the tide of reproductive aging are not elucidated. As is well known, aging in multiple organs is generally accompanied by WNT/β-catenin signaling deficiency, leading to the development of diverse diseases in various tissues including the ovary. In this study, we noticed with interest that multiple mouse models exhibited reduced oocyte quality accompanied by altered WNT/β-catenin signaling, which could be restored by MSC-apoV infusion. In addition, MSC-apoV infusion effectively improved the number of ovarian follicles and oocyte quality, ultimately promoting fertility in 15-month-old aging mice. Notably, while it has previously been reported that infused MSC-apoVs upregulate WNT/β-catenin signaling in the bone and skin 10, 11, we found that MSC-apoVs also downregulated WNT/β-catenin signaling in TCs in this study. Importantly, the mechanisms of apoV infusion on treating ovarian aging are not elucidated enough. The underlying reasons for apoV regulating the transcriptome level of aging ovaries remain to be investigated in future studies. According to the polybasic regulation of apoVs in multiple organs, we suspect that apoVs also possess multidirectional regulation capacity for ovarian homeostasis under different conditions, like apoptotic deficiency and aging. Based on our findings, MSC-apoV infusion may represent a novel, cell-free approach for future reproductive aging therapeutics.

## Materials and Methods

### Animals

All animal experiments were conducted in accordance with the protocol approved by the Institutional Animal Care and Use Committee of Sun Yat-sen University (SYSU-IACUC-2023-000098). 8-week-old and 15-month-old female C57BL/6J mice were purchased from Laboratory Animal Center of Sun Yat-Sen University and GemPharmatech (Nanjing, China). Female C3MRL-Fas*^lpr^*/J (*Fas^mut^*) mice, B6Smn.C3-Fasl^gld^/J (*FasL^mut^*) mice, *Gli1tm3(cre/ERT2)Alj*/J and B6.Cg-*Gt(ROSA)26Sortm9(CAG-tdTomato)Hze*/J (*Gli1-tdTomato*) mice were purchased from the Jackson Laboratory. *CYP17A1-Cre* mice specifically express Cre recombinase in ovarian TCs, which were purchased from Cyagen Biosciences. *Axin1^fl/fl^* mice are placed flox sequences on both sides of *Axin1* sequences, which were donated by Research Center for Human Tissues and Organs Degeneration, Shenzhen Institute of Advanced Technology, Chinese Academy of Sciences. Age-matched 6- to 8-week-old female mice with the same background were used in all experiments.

### PCOS modeling and therapy

To establish the PCOS model, 8-week-old female mice received daily subcutaneous injections of 6 mg/100 g body weight of dehydroepiandrosterone (DHEA, HY-14650, MCE), dissolved in 0.1 mL sesame oil (S27343, YuanYe), for 21 days as the PCOS group; mice injected with an equal amount of sesame oil served as the control group 35. ApoVs in filtered PBS and were injected into PCOS mice through the tail vein at 24 h after the final DHEA injection, once a week for two weeks. Control mice were injected with an equal amount of PBS.

### Morphological observation and quantification of ovarian follicles

After sacrificing the mice, ovarian tissues were isolated and fixed in 4% paraformaldehyde (PFA) (BL539A, Biosharp) overnight. Then, samples were dehydrated by gradient ethanol and embedded in paraffin, and 5-8 μm serial sections were prepared (RM2235, Leica, Germany). Sections then underwent hematoxylin and eosin (H&E, G1080&G1100, Solarbio) staining.

Ovarian follicles were defined according to a previous study 68. Briefly, follicles with more than one layer of granulosa cells without visible antrum were defined as secondary follicles (SF). Follicles with an antral space and a rim of cumulus cells were defined as antral follicles (AF).

### Isolation of ovarian theca cells, mural granulosa cells, cumulus cells and oocytes

Theca cells (TCs) and mural granular cells (MGCs) were isolated and cultured as reported previously 24, 69. Briefly, ovarian tissues from mice were gently separated and put into a 60 mm culture dish (430166, Corning) with MEM Alpha basic (α-MEM, 11380037, ThermoFisher). After puncturing with an insulin needle then mincing under a microscope, mural granular single-cell suspensions were obtained by passing the cells through a 70 μm strainer (352350, Falcon). The rest of ovarian tissue was digested with solution containing 2 mg/mL collagenase type I (LS004197, Biofiven) and 4 mg/mL dispase II (4942078001-1, Roche) in phosphate-buffered saline (PBS) for 1 h at 37 °C. Theca single-cell suspensions were obtained by passing the digested ovarian tissue through a 70 μm strainer. Cumulus cells (CCs) were produced by the microsurgical extirpation of the cumulus oocytes complexes (COCs). GV oocytes were obtained in the ovaries after 48 h administration of pregnant mare serum gonadotropin (PMSG, M2620, Nanjing Aibei Biotechonology Co.) After 48 h of 10 IU PMSG intraperitoneal injection, meiosis II (MII) oocytes were obtained from the fallopian tube after 14 h of 10 IU human chorionic gonadotropin (hCG, M2520, Nanjing Aibei Biotechonology Co.) intraperitoneal injection. TCs and MGCs as well as CCs were seeded on 60 mm culture dishes in complete media containing α-MEM supplemented with 20% fetal bovine serum (FBS, FSP500, ExCell Bio), 2 mM L-glutamine (25030081, Gibco), 100 U/mL penicillin, and 100 μg/mL streptomycin (15140163, Invitrogen), followed by an initial incubation at 37 °C and 5% CO_2_. For NPPC/cGMP/PDE3A/cAMP experiments, MGCs were treated with 1 mM LiCl for 24 h and CCs and oocytes were treated with 0.5 μM XAV939 for 24 h.

### RNA sequencing (RNA-seq) analysis

Ovarian tissues were harvested and washed with PBS for 3 times. Total RNA was isolated from ovarian tissues using Trizol reagent according to the manual instruction. RNA quality was determined by examining A260/A280 with NanodropTM OneCspectrophotometer (Thermo Fisher Scientific Inc). RNA sequencing libraries were generated with an insert size ranging from 200 to 500 bp, and sequenced using the DNBSEQ-T7 sequencer. Raw sequencing data was first filtered by Trimmomatic (version 0.36), low-quality reads were discarded and the reads contaminated with adaptor sequences were trimmed. Clean data were mapped to the reference genome of *mus musculus* using STRA software (version 2.5.3a) with deault parameters. Reads mapped to the exon regions of each gene were counted by featureCounts (Subread-1.5.1; Bioconductor) and then RPKMs were calculated. Genes differentially expressed between groups were identified using the edgeR package (version 3.12.1). A p-value cutoff of 0.05 and fold-change cutoff of 2 were used to judge the statistical significance of gene expression differences. GO analysis and KEGG enrichment analysis for DEGs (Log_2_(fold change) > 1.0 and < -1.0 or Log_2_(fold change) > 1.8 and < -1.8) were both implemented by website of DAVID (DAVID Functional Annotation Bioinformatics Microarray Analysis (ncifcrf.gov)). The data was visualized using website of Bioinformatics platform (https://www.bioinformatics.com.cn/). RNA-seq data output was listed in Table S1.

### Proteomic re-analysis

As described by Zhang *et al*., proteins that were significantly upregulated in apoVs (Fold change > 2 and adjusted p-value < 0.05) were included for further functional analysis based on KEGG and GO databases 5.

### Induction and examination of apoptosis

For apoptosis induction, the MSCs were washed twice with 0.1 μm-filtered PBS (C10010500BT, Thermofisher), and α-MEM (5 mL) with STS (500 nM, ALX-380-014-M005, Enzo life sciences) was added into the dish to induce apoptosis for 6 h. Apoptotic MSCs were observed by optical microscopy (Axio Observer 5, Zeiss). Apoptotic rate of MSCs was detected by terminal deoxynucleotidyl transferase dUTP nick end labeling (TUNEL, G3250, Promega).

### Isolation and characterization of apoVs

ApoVs were isolated by sequential centrifugation according to the protocol reported previously 5, 11. Briefly, the apoptotic MSCs were collected, then cell debris was removed by centrifugation at 800 g for 10 min at 4 °C and 2000 g for 10 min at 4 °C. Subsequently, the supernatant was collected and centrifuged at 16,000 g for 30 min at 4 °C. ApoVs were infused with heparin to protect coagulation effects through the tail vein. Quantification of apoVs was performed using nanoparticle tracking analysis (NTA, Zataview, Particle metrix) for investigation of size and Zata potential with NTA software (Zataview, Particle metrix). ApoVs were stained with CD9 (sc-13118, 1:100), CD63 (sc-5275, 1:100), and CD81 (ab125011, 1:100) for flow cytometry examination (ACEA, NovoCyte).

### Labeling of apoVs

ApoVs were labeled using previously reported methods 10. For labeling of apoVs by membrane dyes, PKH26 (PKH26PCL, Sigma-Aldrich), CellMask™ Deep Red (C10046, Invitrogen) or Annexin V (640908, Biolegand) was used according to the manufacturers' instructions. For labeling of apoVs by AIEgen, DCPy was synthesized according to our established protocol 23. DCPy was then added to serum-free cell culture medium at a final concentration of 5 μM and incubated for 30 min followed by washes. Cells were then induced to undergo apoptosis by light irradiation. ApoVs were infused with heparin to protect coagulation effects through the tail vein. All mice were sacrificed and sampled at 24 h after apoV injection.

### Enzyme linked immunosorbent assay (ELISA)

The levels of plasma testosterone and estradiol (E_2_) were measured using murine ELISA kits (CSB-E05109m and CSB-E05101m, Cusabio). Briefly, 50 μL standard of E_2_ or T at concentrations of 0, 50, 120, 250, 500, 1200 or 0, 1, 2.5, 5, 10, 15 ng/mL or diluted mouse plasma were added to antibody precoated microtest wells. Then, 50 μL of HRP-conjugate mixed solution was added to microtest wells. Next, 96 wells were mixed well and incubated for one hour at 37 °C, then 50 μL of substrate A and 50 μL of substrate B were added to each well after washing three times, then left to incubate for 15 minutes at 37 °C. 50 μL of stop solution was added to each well for 10 min, using a microplate reader set to 450 nM. Concentrations of natriuretic peptide precursor C (NPPC), cGMP, phosphodiesterase 3A (PDE3A), and cAMP were also determined using ELISA kits (JM-02575M2, JM-12746M2, JM-02304M2, Jinmei; ab65355, Abcam) according to the manufacturer's instructions.

### Mating trials

A total volume of 200 μL of solution containing apoVs from 1 × 10^6^ MSCs was prepared and injected them into *Fas^mut^* and *CYP17A1-Cre; Axin1^fl^*^/fl^ and aging mice *via* the tail vein once a week for four weeks. The number of offspring per female was recorded.

### Immunofluorescence (IF) staining

For IF staining, ovarian tissues were isolated and fixed in 4% PFA overnight. The samples were dehydrated in 30% sucrose solution and then embedded in Optimum cutting temperature (OCT, 4583-12, Sakura) compound. Tissue sections were washed with PBS and incubated with 0.3% Triton X-100 for 10 min, then were blocked with 5% BSA for 1 h. Sections were then were incubated with CYP17A1 (94004S, CST), active-β-catenin (05-665, Millipore), DKK1 (AF4600, Affinity), NPPC (Bs-1069R, Bioss), FOXL2 (NB100-1277, Novus), and PPAR-γ (SC7273, Santa cruz) primary antibodies at 4 °C overnight, followed by washing in PBST (PBS+0.1% Triton X-100) and incubation with secondary antibodies and DAPI for 2 h at room temperature, then sections were washed and mounted for confocal imaging. All confocal images were captured on LSM 980 NLO/Zeiss.

ApoVs were co-culture with TCs at cell climbing sheets (whb-24-cs, WHB) for 24 h. TCs were washed and then fixed in 4% PFA for 1 h. TCs were blocked with 5% BSA for 1 h and were stained RNF43 (bs-7007R, Bioss), FZD4 (145901, Thermofisher), cell mask (C10046, Invitrogen), DAPI (C1005, Beyotime) or hoechst (H3570, Invitrogen) primary antibodies at 4 °C overnight. Then, TCs were incubated with secondary antibodies for 1 h and mounted in DAPI mounting medium for confocal imaging. All confocal images were captured on LSM 980 NLO/Zeiss.

Oocytes were isolated and fixed in 4% PFA for 30 min and subsequently incubated with 0.1% Triton X-100 for 10 min. Oocytes were blocked with 5% BSA for 1 h and incubated with active-β-catenin (05-665, Millipore) primary antibodies at 4 °C overnight. Then, oocytes were incubated with secondary antibodies for 1 h. These oocytes were washed with PBS and mounted in DAPI mounting medium (ab104139, abcam) for fluorescence microscope imaging.

### Western blot assay

The total protein from cells or apoVs was harvested and lysed in RIPA (Sc-24948A, Santa Cruz) on ice for 30 min. Total protein concentrations were measured by a BCA protein assay kit (23225, Invitrogen). Western blotting assay was performed using protocols described below. Briefly, total protein (20 μg) was loaded onto 4%-12% NuPAGE™ Bis-Tris gel (NP0321BOX, Invitrogen) and transferred to 0.2 μm polyvinylidene fluoride (PVDF) membranes (ISEQ00010, Millipore). The membranes were blocked with 5% BSA for 1 h at room temperature, then incubated at 4 °C overnight with the primary antibodies against active-β-catenin (05-665, Millipore), β-catenin (9582S, CST), RNF43(bs-7007R, Bioss), GAPDH (5174S, CST), or β-actin (A5441, Sigma). After washing with Tris buffered saline containing 0.1% Tween-20 (TBST), the membranes were incubated at room temperature with species-related HRP-conjugated secondary antibodies (Santa Cruz) diluted at 1:10,000 for 1 h. The immunoreactive proteins were visualized using SuperSignal™ West Pico PLUS Chemiluminescent Substrate kit (34580, Thermo Scientific) and SuperSignal™ West Femto Maximum Sensitivity Substrate kit (34095, Invitrogen), and detected by a ChemiDoc™ MP imaging system (Bio-Rad, USA).

### Flow cytometry analysis

To examine the RNF43 expression, MSCs and MSC-derived apoVs as well as circulating apoVs from organisms were treated with RNF43 primary antibody (bs-7007R, Bioss) for 1 h at 4 ºC at a concentration of 1:200 in filtered PBS. After centrifugation and washing with PBS, MSCs and MSC-derived apoVs as well as circulating apoVs were stained with FITC-conjugated donkey anti-rabbit secondary antibody (406403, Biolegend) for 1 h at 4 ºC at a concentration of 1:200 in filtered PBS. To examine the relative MSC marker expression of TCs, TCs were stained with PE anti-mouse CD34 (551387, BD), PE anti-mouse CD45 (12-0451-82, Invitrogen), PE anti-mouse CD44 (553134, BD), PE anti-mouse CD73 (550741, BD), PE anti-mouse CD90 (554898, BD), PE anti-mouse CD105 (562759, BD), and PE anti-mouse Sca-1 (108108, Biolegand) for 1 h at 4 ºC at a concentration of 1:200 in filtered PBS. To characterize apoVs, apoVs were stained with PE anti-mouse CD63 (143903, Biolegand), PE anti-mouse CD81 (104905, Biolegand), or APC anti-mouse TSP1 (15571-05061, Sytan) for 1 h at 4 ºC at a concentration of 1:200 in filtered PBS.

### Nanoflow cytometry

According to the manufacturer's protocol, nanoflow cytometry (Flow NanoAnalyzer, NanoFCM Inc.) was used to analyze circulating apoVs stained with Annexin V in WT, *Fas^mut^,* and *FasL^mut^* mice 70. Particle concentration and size distribution were calculated using the NanoFCM software (NanoFCM Profession V1.0).

### TUNEL assay

The apoptotic MSCs were fixed with 4% PFA and permeabilized. According to the manufacture's protocol, cells were incubated with TUNEL reagent (G3250, Promega) for 60 min at 37 ºC. Positive cells were counted after counterstaining with DAPI under a microscope.

### Kidney capsule implantation

About 1 × 10^6^ mouse TCs or GCs at passage 1 were implanted underneath the kidney capsule of recipient mice. Transplantation of mice TCs or GCs were performed as previously reported 71. Briefly, 1× 10^6^ TCs or GCs were mixed and placed under the kidney capsule of C57BL/6J mice. Mice were euthanized 2 months after implantation.

### Statistical analysis

Data are presented as mean ± SD of at least triplicate measurements. Statistical significance was evaluated by two-tailed Student's unpaired *t* test for two-group comparison. Comparisons between more than two groups were analyzed using one-way ANOVA. *P* values less than 0.05 were considered statistically significant. Graph analysis was performed using GraphPad Prism 8.00 (GraphPad Software, USA).

### Data availability

All relevant data and code are available within the article and its supplementary information/Source data or freely available from the corresponding authors upon reasonable request.

## Supplementary Material

Supplementary figures.

Supplementary table 1.
