# Supplementary material for: Mesenchymal stem cell-derived apoptotic vesicles ameliorate impaired ovarian folliculogenesis in polycystic ovary syndrome and ovarian aging by targeting WNT signaling
Source: Theranostics. 2024 May 27;14(8):3385–403. doi: 10.7150/thno.94943 (PMC11155401; doi:10.7150/thno.94943)
Supplement: Supplementary file 1 — Supplementary figures. [file thnov14p3385s1.pdf]

Mesenchymal stem cell-derived apoptotic vesicles ameliorate impaired ovarian folliculogenesis in polycystic ovary syndrome and ovarian aging by targeting WNT signaling

Yu Fu, Manjin Zhang, Bingdong Sui, Feifei Yuan, Wenbo Zhang, Yashuang Weng, Lei Xiang, Can Li, Longquan Shao, Yong You, Xueli Mao, Haitao Zeng, Di Chen, Meijia Zhang, Songtao Shi*, Xuefeng Hu*

* Correspondence to: bioxfh@fjnu.edu.cn (Xuefeng Hu) and shisongtao@mail.sysu.edu.cn (Songtao Shi)

This file includes:

Extended Data Figures. S1 to 6, with their legends

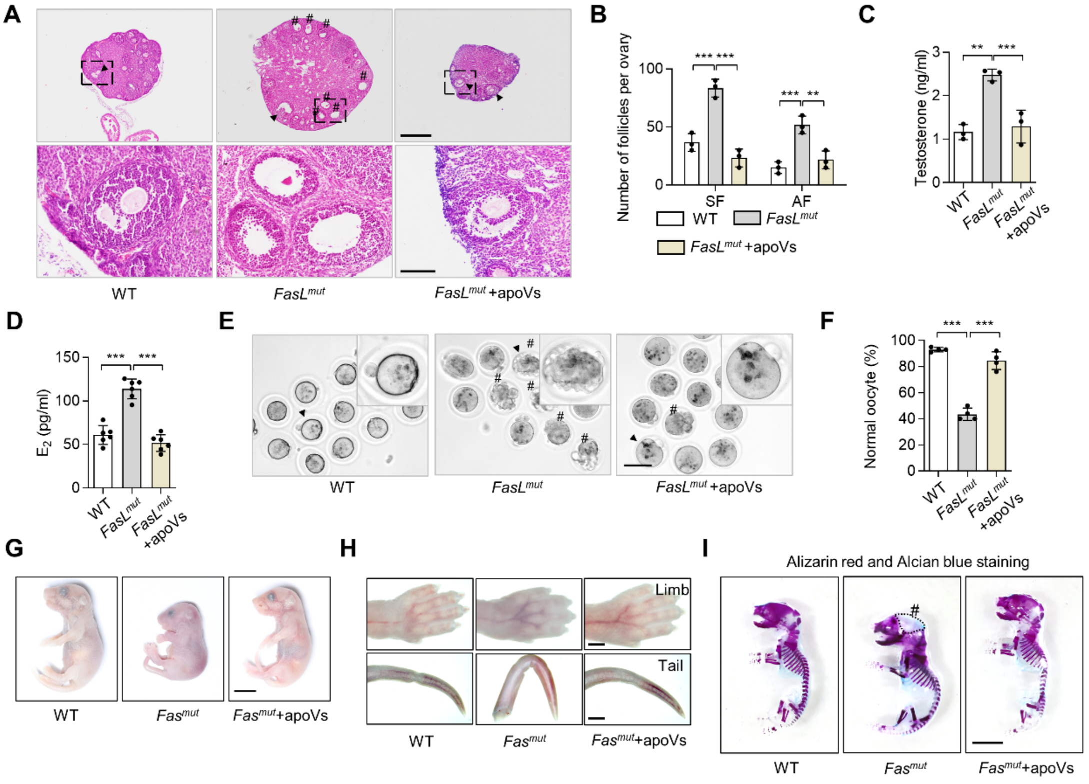

Figure S1. MSC-apoV infusion ameliorate apoptotic deficiency-induced ovarian dysfunction. (**A**) Representative H&E staining of ovarian tissues. Lower panels are higher magnification views of the boxed regions in the above H&E staining images. Black arrowheads indicate AF. Pound signs indicate polycystic-like follicles. Scale bars, 500 μm for lower magnification, 100 μm for higher magnification. (**B**) The numbers of SF and AF in the ovaries. *N* = 3. (**C**) ELISA analysis showing the levels of testosterone in serum. *N* = 3. (**D**) ELISA analysis showing the levels of E_2 in serum. *N* = 6. (**E**) Gross appearance of MII oocytes. Pound signs indicate abnormal oocytes. Black arrowheads indicate higher magnification views. Bar = 50 μm. (**F**) The ratio of normal MII oocytes/total oocytes. *N* = 4. (**G**) Gross appearance of fetuses. Scale bar, 0.5 cm. (H) Gross appearance of fetal limbs and tails. Bar = 1 mm. (**I**) Alcian blue and Alizarin red staining of skeletons. Error bars are means ± SD. Data were analyzed using one-way analysis of variance (ANOVA) with Tukey's test. $^{**}P < 0.01$, $^{***}P < 0.001$.

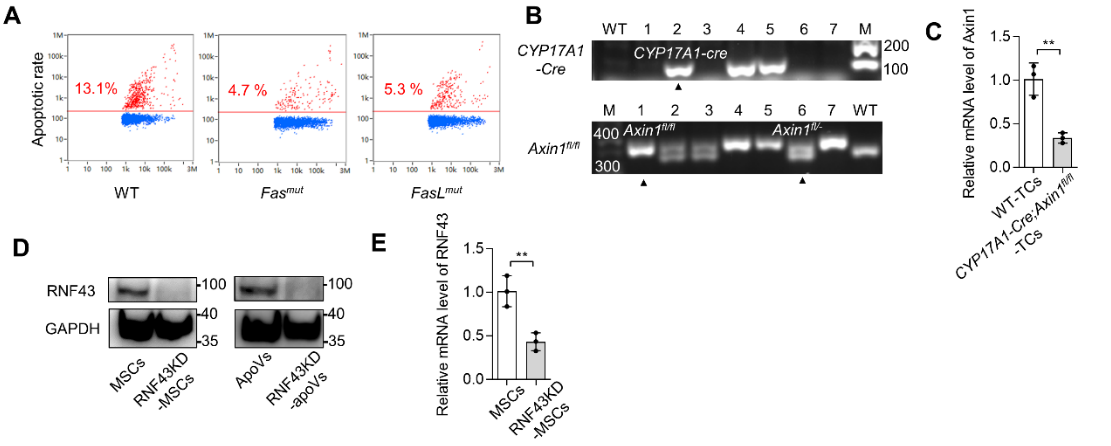

Figure S2. Identification of transgenic mice and RNF43 knockdown in apoVs. (**A**) Nano flow cytometry analysis showing apoptotic ratios of circulating apoVs in WT, Fas mutant (*Fas*^{mut}) and Fas ligand mutant (*FasL*^{mut}) mice. (**B**) Genotyping of *CYP17A1-Cre; Axin1*$^{fl/fl}$ mice. (**C**) mRNA examination of *Axin1* in ovarian TCs of *CYP17A1-Cre; Axin1*$^{fl/fl}$ mice. (**D**) Western blot analysis confirmed RNF43 levels in MSCs and RNF43 knockdown (RNF43KD)-MSCs as well as apoVs and RNF43-KD apoVs. (**E**) mRNA examination of RNF43 in MSCs. Data were analyzed using two-tailed Student's unpaired *t* test for two-group comparison. *P < 0.05, **P < 0.01, ***P < 0.001.

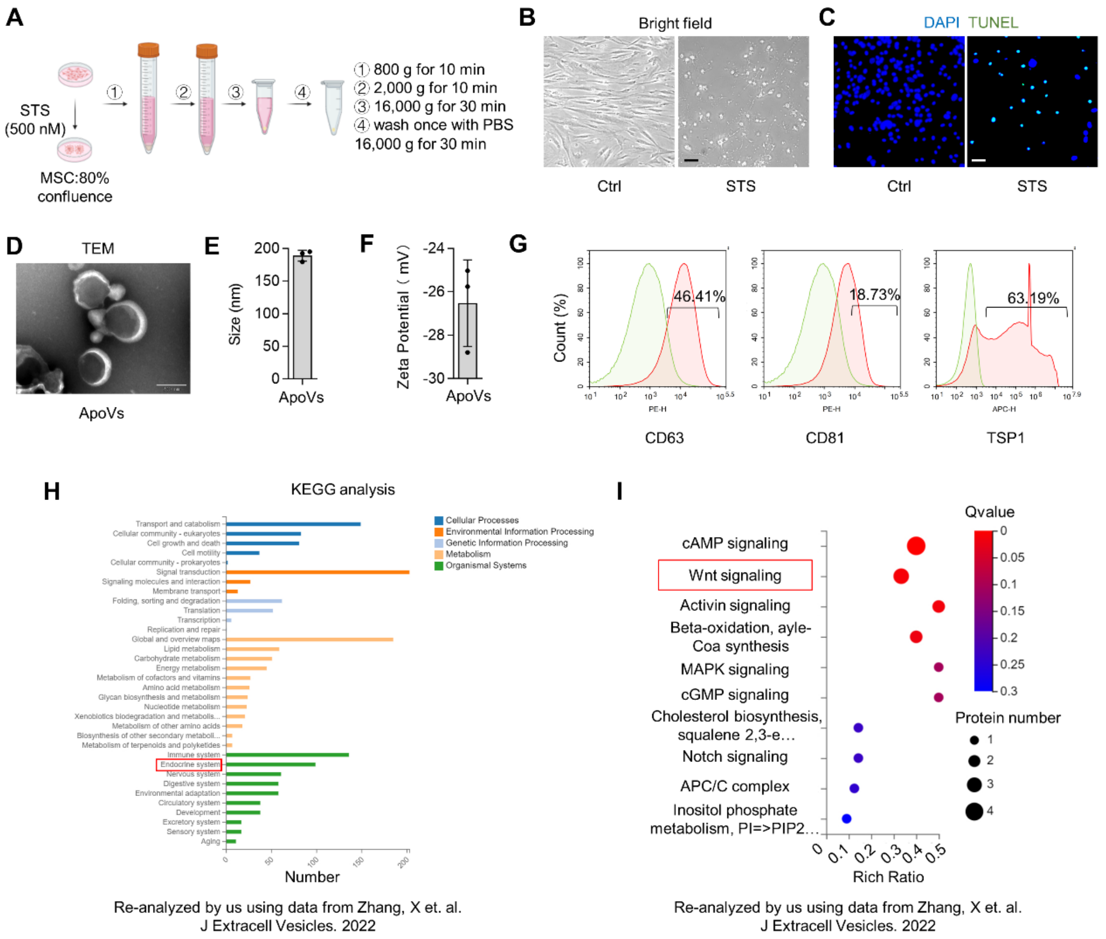

Figure S3. Characterization of MSC-apoVs. (**A**) Schematic illustration of protocol for isolating apoVs from apoptotic MSCs. (**B**) MSC morphology and morphological changes after 6 h of STS induction observed under microscopy. Scale bar, 50 μm. (**C**) Detection of cell apoptosis using TUNEL staining. Scale bar, 50 μm. (**D**) Representative TEM images of apoVs. Scale bar, 500 nm. (**E** and **F**). The size and electric potential of apoVs in 1 × PBS buffer (pH 7.4). N = 3. (**G**) Flow cytometry analysis of apoV markers CD63, CD81 and TSP1. (**H**) Re-analysis of proteomic results, KEGG pathway of significantly upregulated proteins in MSC-apoVs. (**I**) Re-analysis of KEGG pathway from (**E**) “Endocrine system”.

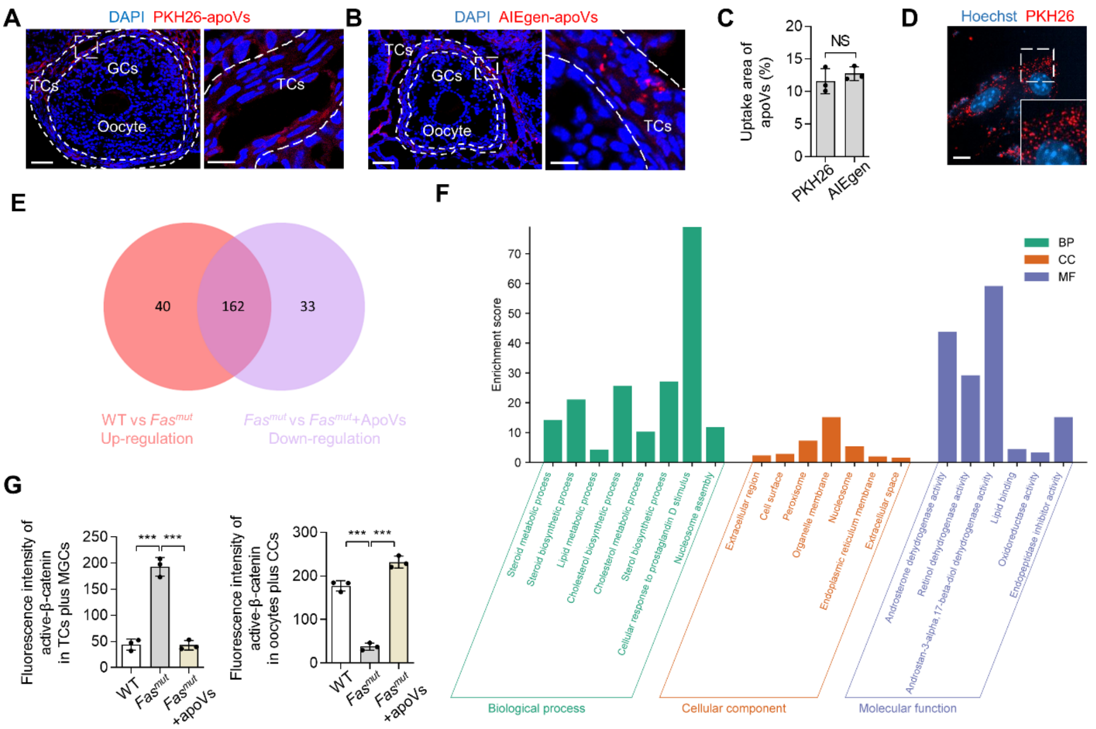

Figure S4. Systemically infused MSC-apoVs are predominantly endocytosed by ovarian theca cells and regulate the steroid metabolic process of ovary. (**A** and **B**) Tracing of PKH26-labeled and AIE-gen-labeled MSC-apoVs (red) in the ovarian follicles. Right panels are higher magnification views of the left boxed regions. White dashed lines indicate ovarian theca cell (TC) regions. Scale bars, 50 μm for lower magnification, 10 μm for higher magnification. (**C**) Quantification of ratio of cell uptake area of MSC-apoVs/total area of whole ovary. *N* = 3. (**D**) PKH26-labeled MSC-apoVs co-cultured with TCs for tracing MSC-apoV location in TCs *in vitro*. Higher magnification shows apoVs were mainly enriched in the perinuclear area. Bar = 10 μm. (**E**) Venn diagram showing the number of up-regulated gene (WT vs *Fas*^{mut}) and down regulated gene (*Fas*^{mut} vs *Fas*^{mut} + apoVs, Log$_2$(fold change) > 1.8 or < -1.8) (**F**) GO analysis of the intersection of up-regulated gene (WT vs *Fas*^{mut}) and down regulated gene (*Fas*^{mut} vs *Fas*^{mut} + apoVs, Log$_2$(fold change) > 1.8 or < -1.8). (**G**) Quantification of fluorescence intensity of active β-catenin (green) in TCs plus MGCs and oocytes plus CCs. *N* = 3.

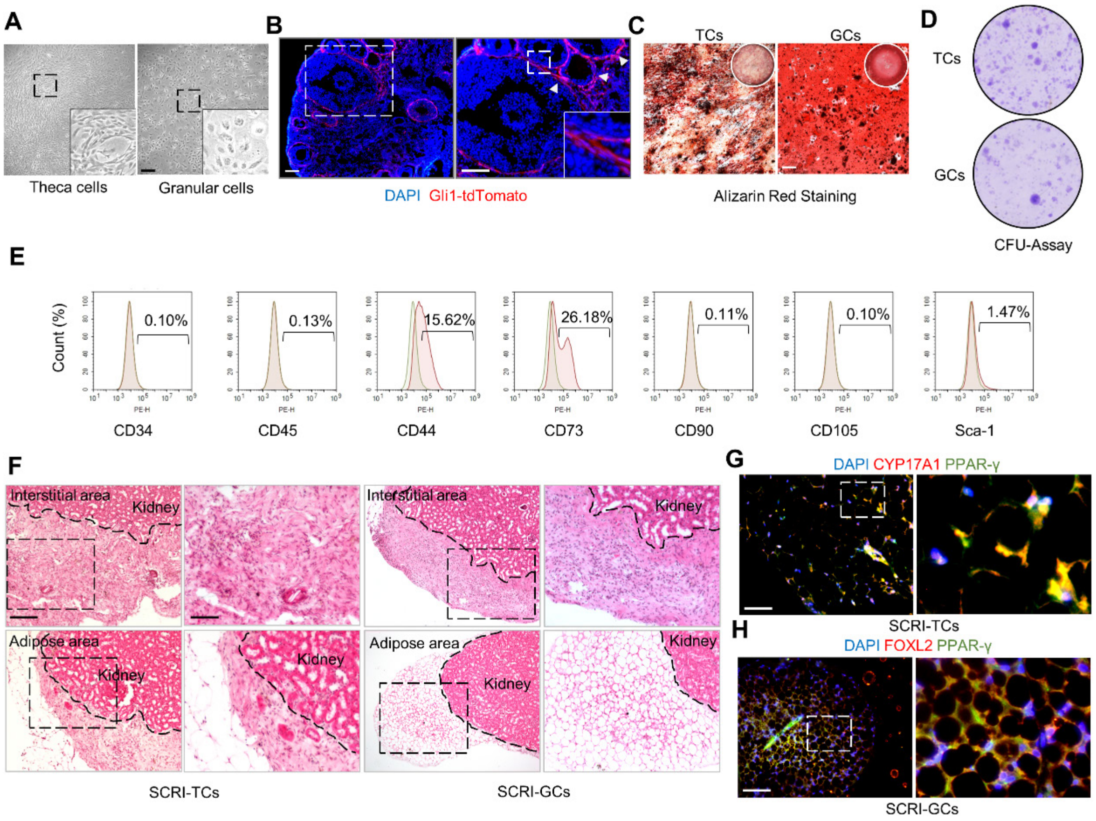

Figure S5. Ovarian theca cells possess MSC characteristics. (**A**) Theca cell (TC) and granular cell (GC) morphology under bright field microscopy. Higher magnification views of the boxed regions are shown in insets. Scale bar, 200 μm. (**B**) Immunofluorescent staining of MSC marker Gli1 in Gli1-tdTomato mice. Right panels are higher magnification views of the boxed regions in the left images. White arrowheads indicate Gli1-positive regions. Scale bar, 200 μm. (C) Osteogenic differentiation capacity of TCs and GCs detected by alizarin red staining. Scale bar, 200 μm. (**D**) Colony-Forming-Unit (CFU) assay analysis of TCs and GCs. (**E**) Flow cytometry analysis of MSC-specific markers, including negative markers CD34 and CD45; positive markers CD44, CD73, CD90, CD105, and Sca-1. (**F to H**) Representative H&E and immunofluorescence images of kidney capsule implantation of TCs and GCs in C57BL/6 mice. Right panels are higher-magnification views of the boxed regions in the left H&E and immunofluorescence images. Scale bar, 500 μm.

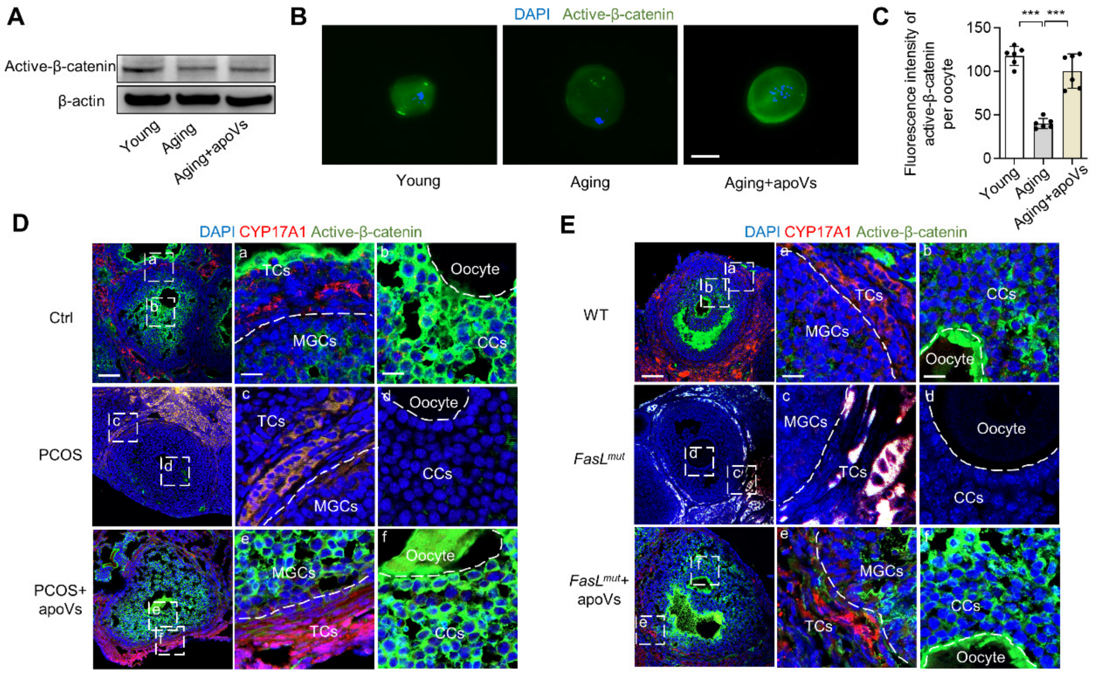

Figure S6. WNT/β-catenin down-regulation in oocytes of PCOS and aging mice. (**A**) Western blot analysis showing active β-catenin levels in the whole ovary, indicating WNT signaling activity. (**B**) Immunofluorescent staining of active β-catenin level in GV oocytes. Bar = 50 μm. (**C**) Quantification of fluorescence intensity of active β-catenin per oocyte showing significant differences. *N* = 6. (**D**) Immunofluorescent co-staining of TC-specific marker CYP17A and active β-catenin in the ovaries of PCOS and MSC-apoV-treated PCOS mice. Right panels are higher magnification views of the left boxed regions, highlighting TC and MGC regions (a, c, e), as well as oocyte and CC regions (b, d, f). White dashed lines are used to divide cells in different regions. Scale bars, 50 μm for lower magnification, 10 μm for higher magnification. (**E**) Immunofluorescent co-staining of TC-specific marker CYP17A1 and active β-catenin in the ovaries of *FasL*^{mut} and MSC-apoV-treated *FasL*^{mut} mice. Right panels are higher magnification views of the left boxed regions, highlighting TC and MGC regions (a, c, e), as well as oocyte and CC regions (b, d, f). White dashed lines are used to divide cells in different regions. Scale bars, 50 μm for lower magnification, 10 μm for higher magnification. Error bars are means ± SD. Data were analyzed using one-way analysis of variance (ANOVA) with Tukey's

test. NS, not significant. *P < 0.05, **P < 0.01, ***P < 0.001.
